# Parvalbumin neuroplasticity compensates for somatostatin impairment, maintaining cognitive function in Alzheimer’s disease

**DOI:** 10.1186/s40035-022-00300-6

**Published:** 2022-05-03

**Authors:** Christopher Daniel Morrone, Aaron Yenhsin Lai, Jossana Bishay, Mary Elizabeth Hill, JoAnne McLaurin

**Affiliations:** 1 Biological Sciences, Sunnybrook Research Institute, Toronto, ON M4N 3M5 Canada; 2 Department of Laboratory Medicine and Pathobiology, Temerty Faculty of Medicine, University of Toronto, Toronto, ON M5S 1A8 Canada

**Keywords:** Alzheimer’s disease, TgF344-AD rat, GABAergic interneuron, Parvalbumin, Somatostatin, Barnes maze, Hippocampus, Neuronal compensation, Cognitive resilience

## Abstract

**Background:**

Patient-to-patient variability in the degree to which β-amyloid, tau and neurodegeneration impact cognitive decline in Alzheimer’s disease (AD) complicates disease modeling and treatment. However, the underlying mechanisms leading to cognitive resilience are not resolved. We hypothesize that the variability in cognitive function and loss relates to neuronal resilience of the hippocampal GABAergic network.

**Methods:**

We compared TgF344-AD and non-transgenic littermate rats at 9, 12, and 15 months of age. Neurons, β-amyloid plaques and tau inclusions were quantified in hippocampus and entorhinal cortex. Somatostatin (SST) and parvalbumin (PVB) interneurons were traced to examine hippocampal neuroplasticity and cognition was tested in the Barnes maze.

**Results:**

The 9-month-old TgF344-AD rats exhibited loss of neurons in the entorhinal cortex and hippocampus. Hippocampal neuronal compensation was observed in 12-month TgF344-AD rats, with upregulation of GABAergic interneuronal marker. By 15 months, the TgF344-AD rats had robust loss of excitatory and inhibitory neurons. β-Amyloid and tau pathology accumulated continuously across age. SST interneurons exhibited tau inclusions and atrophy from 9 months, whereas PVB interneurons were resilient until 15 months. The hippocampal PVB circuit underwent neuroplastic reorganization with increased dendritic length and complexity in 9- and 12-month-old TgF344-AD rats, before atrophy at 15 months. Strikingly, 12-month-old TgF344-AD rats were resilient in executive function and cognitive flexibility. Cognitive resilience in TgF344-AD rats occurred as maintenance of function between 9 and 12 months of age despite progressive spatial memory deficits, and was sustained by PVB neuroplasticity.

**Conclusions:**

Our results demonstrate the inherent neuronal processes leading to cognitive maintenance, and describe a novel finding of endogenous cognitive resilience in an AD model.

**Supplementary Information:**

The online version contains supplementary material available at 10.1186/s40035-022-00300-6.

## Background

Alzheimer’s disease (AD) progression involves deposition of β-amyloid (Aβ) plaques, neurofibrillary tangle deposits of hyperphosphorylated tau, and neurodegeneration most prominently in tau-affected brain regions [1–3]. Once these pathologies accumulate beyond a threshold, patients present with clinically relevant cognitive dysfunction, including loss of spatial memory and executive function [1, 4]. A large focus of current AD research is on Aβ and tau therapeutics; however, patient-to-patient variability in the threshold to which these pathologies impact cognition complicates clinical translation.

Emerging evidence demonstrates that the excitatory/inhibitory balance maintains cognitive function, and an imbalance links AD pathology with cognitive decline [5]. The GABAergic interneurons have an adaptive role in sustaining this balance [6–8]. Notably, somatostatin (SST) and parvalbumin (PVB) cells account for ~ 70% of interneurons, and provide dendritic and somatic inhibition, respectively, to regulate excitatory activity and cognition [6, 9, 10]. SST and PVB interneurons exhibit varied responses to AD pathology across disease progression, including neurodegeneration and dendritic remodelling [11–14]. Furthermore, recent evidence demonstrates that accumulation of tau within PVB and SST neurons impairs hippocampal neurogenesis [15–17]. Although the role of GABAergic interneurons in cognitive function is undisputed, their contribution to neuronal and cognitive resilience across AD progression has not been fully elucidated.

Cognitive reserve refers to the variability between individuals to withstand greater degrees of neurodegeneration before exhibiting cognitive impairment [16]. AD patients at a comparable cognitive stage often exhibit differing Aβ and tau loads [16–18]. Neuronal compensation, including greater number of neurons and neuroplastic circuit reorganization, sustains cognitive reserve [17, 19]; however, the contribution of these mechanisms to cognitive reserve in AD has not been elucidated, as it is a difficult process to investigate in preclinical disease models.

In this study, we utilized the TgF344-AD rat model as it recapitulates Aβ pathology, hyperphosphorylation and deposition of tau into neuritic and pre-tangle inclusions, and neurodegeneration including neuronal loss [20–24]. Notably, tau pathology in TgF344-AD rats occurs in response to Aβ-induced neuronal damage, and is composed of physiologically-expressed, endogenous rat tau that is hyperphosphorylated and aggregates in patterns akin to Braak staging [20, 21, 25]. In TgF344-AD rats, the locus coeruleus tau precedes entorhinal cortical and hippocampal deposition [21], and tau progresses more rapidly in the entorhinal cortex than in the hippocampus, at early-to-mid disease stages [24], as we further demonstrate here. Tau pathology without genetic mutation or overexpression is an additional advantage in examining its impact on inhibitory and excitatory neurons across AD progression. Critically, TgF344-AD rats are an advantageous AD model, recapitulating core features of disease progression, most importantly in the switch of endogenous, healthy tau protein into a pathological form, becoming a driving force behind neurodegeneration and cognitive impairments [20, 24].

Aβ and tau deposit from 6 months in TgF344-AD rats [20, 21], with extensive accumulation and neurodegeneration by 9 months of age [22, 23]. Behaviorally, TgF344-AD rats exhibit robust impairments in spatial, recognition and working memory, as well as executive function beginning at 13–15 months of age [20, 24, 26, 27]. However, there are reports of earlier cognitive disruption in working memory [27, 28] and in executive function at as early as 6 months of age [21]. In light of these findings, we propose that the TgF344-AD rats are an important rodent model to investigate contributions of neuronal compensatory mechanisms to cognition across AD progression.

## Materials and methods

### Experimental model: TgF344-AD rats and non-transgenic littermates

All animal experiments were in accordance with the ethical standards of the Canadian Council on Animal Care guidelines and approved by the Animal Care Committee of the Sunnybrook Research Institute (#20-655). Non-transgenic (NTg) and TgF344-AD rats were outbred on a Fischer strain in-house, and housed on a 12-h light–dark schedule with ad libitum access to chow and water. The TgF344-AD rats recapitulate AD pathology through overexpression of human amyloid precursor protein with the Swedish mutation (KM670/671NL) and presenilin 1 with exon 9 excised, under the mouse prion promotor. Behaviorally naïve, sex-balanced TgF344-AD and age-matched NTg littermate Fischer 344 rats were utilized in this study. The rats were assigned to age groups based on a balance of genotype and sex, across multiple litters: behavior 9- (*n* = 30), 12- (*n* = 26), and 15-months (*n* = 31), as well as 12-month repeat (*n* = 16); pathological assessment 4- (*n* = 6), 9- (*n* = 17), 12- (*n* = 17) and 15-months (*n* = 18). For each experiment, the numbers of individuals per age and genotype are specified in the figure legends.

### Barnes maze

All rats were naïve to behavioral assessment. Barnes maze (Maze Engineers, Boston, MA) testing was conducted in a behavioral suite with spatial cues, with an aversive light in all trials except the training. EthoVision XT (Noldus, Wageningen, Netherlands; version 11.5) was utilized to collect and analyze video data. Following training to the location of the escape, the rats learned the task across 3 days, with 2 trials per day. Spatial memory was assessed 3 days later, in one probe trial. Reversal learning (5 days, 2 trials per day) for executive function began the next day, in which the location of the escape hole was switched without re-training. Rats were assessed for their latency to find the escape hole, and search strategies were manually scored by an investigator blinded to the groups as previously described: direct (1), corrected (0.75), long correction (0.5), focused search (0.5), serial (0.25), and random (0) [24, 29].

### Tissue collection

The NTg and TgF344-AD rats were sacrificed under anesthesia (5% isoflurane) at 9, 12 and 15 months of age for pathological assessment. Brain tissues were collected following transcardial perfusion with phosphate-buffered saline (PBS) and then 4% paraformaldehyde. The rat brains were sectioned with a sliding microtome, and 40-μm sections were collected throughout the hippocampus and entorhinal cortex (bregma − 1.80 to − 8.00 mm).

### Immunohistochemistry for Aβ, tau, and glutamate decarboxylase (GAD67)

Immunohistochemistry was conducted for Aβ (6F3D), GAD67, and tau (PHF1) similar to previously published work [22, 24]. For Aβ and GAD67, 4 sections spaced 1 in 20 were sampled throughout the hippocampus (starting: bregma − 3.00 mm), and 3 sections spaced 1 in 10 throughout the entorhinal cortex (starting: bregma − 5.75 mm). Tau sampling was performed similarly, except with 3 hippocampal and 3 entorhinal sections.

Aβ: All washes were in 1 × PBS, and were conducted between all incubations, unless otherwise stated. Sections were washed and incubated for 30 min with 1% hydrogen peroxide, to block endogenous peroxidase activity. Next, the sections underwent antigen retrieval for 5 min with 70% formic acid. Primary antibody incubation with mouse anti-Aβ (6F3D, 1:400; Dako, Agilent Dako, Burlington, Ontario, Canada; M0872) diluted in PBS with 0.2% bovine serum albumin (BSA) and 0.2% Triton X-100, was conducted overnight at room temperature. The following day, sections were incubated with Vectastain ABC kit anti-mouse secondary antibody (1:400; Vector Laboratories, Burlingame, CA; PK-4002) diluted in PBS with 0.2% BSA and 0.2% Triton X-100, for 1.5 h, followed by incubation with the ABC solution (1:200; Vector Laboratories, PK-4002) diluted in PBS for 1 h. The sections were developed with 3,3'-diaminobenzidine peroxidase substrate kit (Vector Laboratories, SK-4100). Finally, the sections were mounted, dehydrated in ethanol (70%, 95%, 100%) and xylene, and cover-slipped with permount.

GAD67: Staining for GAD67 (mouse anti-GAD67, 1:1000; Millipore Sigma, Darmstadt, Germany; MAB5406) was performed as described above, with the omission of the formic acid antigen retrieval step, and the addition of a 1-h block immediately prior (no wash step) to incubation with primary antibody in PBS with 0.2% BSA, 0.2% Triton X-100 and 2% dimethyl sulfoxide.

Tau: All washes were in tris-buffered saline (TBS) and were conducted between all incubations, unless otherwise stated; 0.05% Triton X-100 was added to the washes following primary incubation until the wash before 3,3'-diaminobenzidine development. Sections were washed and incubated for 30 min with 3% hydrogen peroxide with 0.25% Triton X-100 in TBS, to block endogenous peroxidase activity. Next, the sections were blocked in 5% milk in TBS for 1 h, and transferred to the primary incubation without washing: mouse IgG1 anti-PHF1 (1:1000; courtesy of the late Dr. Peter Davies, The Feinstein Institute for Medical Research) diluted in 5% milk, overnight at 4 °C. The following day, sections were incubated with secondary goat anti-mouse IgG1-biotinXX (1:200, Invitrogen, Waltham, MA; A10519) diluted in 20% superblock (Invitrogen), for 1.5 h, followed by incubation with the ABC solution (1:200; Vector Laboratories, PK-4002) diluted in 20% superblock, for 1 h. The sections were then developed, mounted, dehydrated and cover-slipped as described above for Aβ staining.

All sections were imaged on a Zeiss Observer Z1 microscope: Aβ (10× analysis, 20× representative); tau (20× analysis, 63× representative); GAD67 (10× analysis, 63× representative). The total entorhinal and hippocampal GAD67 cells and tau inclusions were quantified manually and normalized to area (mm^2^). PHF1 inclusions < 10 μm from a plaque were considered plaque-associated; the rest were non-plaque-associated [24, 30]. Analysis of Aβ and tau per plaque was conducted by subtracting background, binarizing images and analyzing staining density (% area covered) in ImageJ (Fiji open-source, version 1.53f51, National Institutes of Health, USA).

### Immunofluorescence for NeuN, SST, and PVB

Immunofluorescence staining was conducted similar to previous descriptions [24]. For neuronal nuclei (NeuN): 4 sections spaced 1 in 20 were sampled throughout the hippocampus (starting: bregma − 3.00 mm), and 3 sections spaced 1 in 10 throughout the entorhinal cortex (starting: bregma − 5.75 mm). Briefly, sections were washed in PBS (NeuN, PVB) or TBS (SST), blocked in 2% (NeuN), 5% (SST) or 10% (PVB) donkey serum in 0.1% (NeuN) or 0.5% (SST, PVB) Triton X-100 containing 1% (NeuN, PVB) or no (SST) BSA, prior to primary incubation in the block solution overnight (guinea pig anti-NeuN, 1:500; Millipore-Sigma, ABN90) or for 3 days (rabbit anti-SST, 1:100; Invitrogen, Waltham, MA; PA5-85759; or mouse anti-PVB, 1:2000; Swant, Burgdorf, Switzerland; PV 235). Following washes, the sections were incubated for 2 h with secondary donkey anti-guinea pig (Alexafluor594, 1:200; Jackson ImmunoResearch Laboratories, West Grove, PA; 706-585-148), donkey anti-rabbit (Alexafluor488, 1:200; Invitrogen, A-21206) or donkey anti-mouse (Alexafluor488, 1:200; Invitrogen, A-21202). For NeuN and PVB (anti-guinea pig and anti-mouse), the secondaries were incubated in the same block solution. For SST (anti-rabbit), the secondary incubation was in 0.5% BSA and 0.5% Triton X-100. Sections were washed, mounted and cover-slipped with PVA-DABCO. NeuN images were collected at 10× on a Zeiss Observer Z1 microscope. Images were processed, binarized and analyzed for staining density (% area covered and number of cells/mm^2^) in ImageJ. Hippocampal SST and PVB images (approximately bregma − 4.00 mm) were collected as z stacks at 20× and 60× (PVB only) on a NikonA1 (Melville, New York, NY) laser scanning confocal microscope. For 9-, 12- and 15-month-old rats, a total of 16 cells per region and per cohort (across *n* of 4 rats) were sampled for SST and PVB dendritic length and complexity analysis, while for 4-month-old rats, 12 cells per region per cohort, across *n* of 3 rats, were analyzed. Neuronal tracing of all dendritic processes for each sampled neuron was conducted via the Simple Neurite Tracer plugin for ImageJ Fiji, followed by the polynomial best fitting degree Sholl analysis [31], and intersections analyzed with intervals of 5- (SST) or 10-μm (PVB) radius from the soma.

### PHF1 co-localization of SST, PVB, NeuN, and glutaminase (GLS)

PHF1 immunofluorescence was performed as previously described (see [24] supplementary material), with the addition of co-labelling for SST, PVB (rabbit anti-PVB, 1:1000; Abcam, Cambridge, United Kingdom; ab11427), NeuN or glutaminase (GLS; rabbit anti-KGA/GAC, 1:250; Proteintech, Rosemont, IL; 12855-1-AP). Briefly, washes were in TBS, or TBS with 0.05% Triton X-100 (after primary incubation). Blocking was in 5% milk in 0.25% Triton X-100 and primary incubations were in 5% milk. For secondary labelling, PHF1 signal was amplified by a 2-h incubation with goat anti-mouse IgG1-biotinXX (1:80, Invitrogen, A10519) in 20% superblock (Invitrogen), followed by washes and then a 2-h incubation with an Alexafluor647-conjugated streptavidin (1:200, Invitrogen, S21374) and either Alexafluor488 donkey anti-rabbit (for detection of SST, PVB, or GLS) or Alexafluor594 donkey anti-guinea pig (for detection of NeuN), diluted together in 20% superblock. Sections were washed, mounted and cover-slipped with PVA-DABCO. Images were collected as z-stacks at 10× (for analysis, hippocampus only) and 60× (representative images) on a NikonA1 laser scanning confocal microscope in the entorhinal cortex and hippocampus. Hippocampal SST^+^/PHF1^+^ and PVB^+^/PHF1^+^ neurons were quantified manually in ImageJ across 3 sections per rat, in both hemispheres, and normalized to total SST^+^ or PVB^+^ cells. The SST^+^ neurons were quantified as plaque-associated (< 10 μm from a plaque) or non-plaque-associated, to determine the percentage of dystrophic cells. All reagents utilized in immunohistochemistry and immunofluorescence experiments are listed in Additional file 1: Table S1.

### Statistical analyses

For behavioral and pathological assessments, we based our number of rats on previously published work in the TgF344-AD model [24]. One outlier was removed from the 15-month behavior cohort: statistical outlier on probe trial (Grubb’s test) and non-compliance during learning trials. There were no other data exclusions. Rats were assigned to sex- and genotype-balanced age groups (at 4, 9, 12 and 15 months of age) across multiple litters per age. Investigators were blinded to genotype during data collection and analysis, prior to statistical analysis. GraphPad Prism 6 and 9 (GraphPad Software, Inc., San Diego, CA) were used for two-sided statistical analysis and generation of graphs. SPSS (IBM, version 23) was used for generation of graphs of search-strategy percentage in the reversal days. Two-way ANOVA with a Holm-Sidak *post-hoc* test was used for all analyses with age and genotype as independent variables. Unpaired *t*-test or Mann–Whitney U test (spatial memory search strategies were not averaged over 2 trials, and were therefore analyzed as ordinal data) was conducted for two-group analysis. Repeated measures ANOVA, with correction for multiple comparisons with a Holm-Sidak *post-hoc* test, was conducted for the Barnes maze learning and reversal trials (genotype, trial day) and for Sholl analysis (genotype, distance from cell soma). One-way and two-way ANOVA with a Holm-Sidak *post-hoc* test, as well as linear regression and non-linear exponential growth regression were conducted for Aβ and tau pathology. One-way ANOVA with a Holm-Sidak *post-hoc* test was conducted for tau co-localization and SST dystrophy analyses. Linear regression was conducted for all behavioural age effects and for behaviour × interneuron correlation analyses. All data are mean ± standard error of the mean (SEM) or ± 95% confidence intervals. Statistical significance was determined by a *P* value equal to or less than 0.05 (exact values reported in the results section). Exact *n*, data type and statistical tests utilized are reported in the figure graphs and legends.

## Results

To model neuronal compensation and loss in AD, we assessed entorhinal cortical and hippocampal excitatory and inhibitory neurons in separate cohorts of sex-balanced rats across age, beginning from a stage with established pathology: 9 months [22, 23]. No sex differences were detected in any of the readout measures examined; therefore, male and female mice were grouped in all readouts, and data reported as effects of age and genotype.

### Age-related entorhinal cortical neuronal loss and hippocampal neuronal compensation

**Fig. 1:**
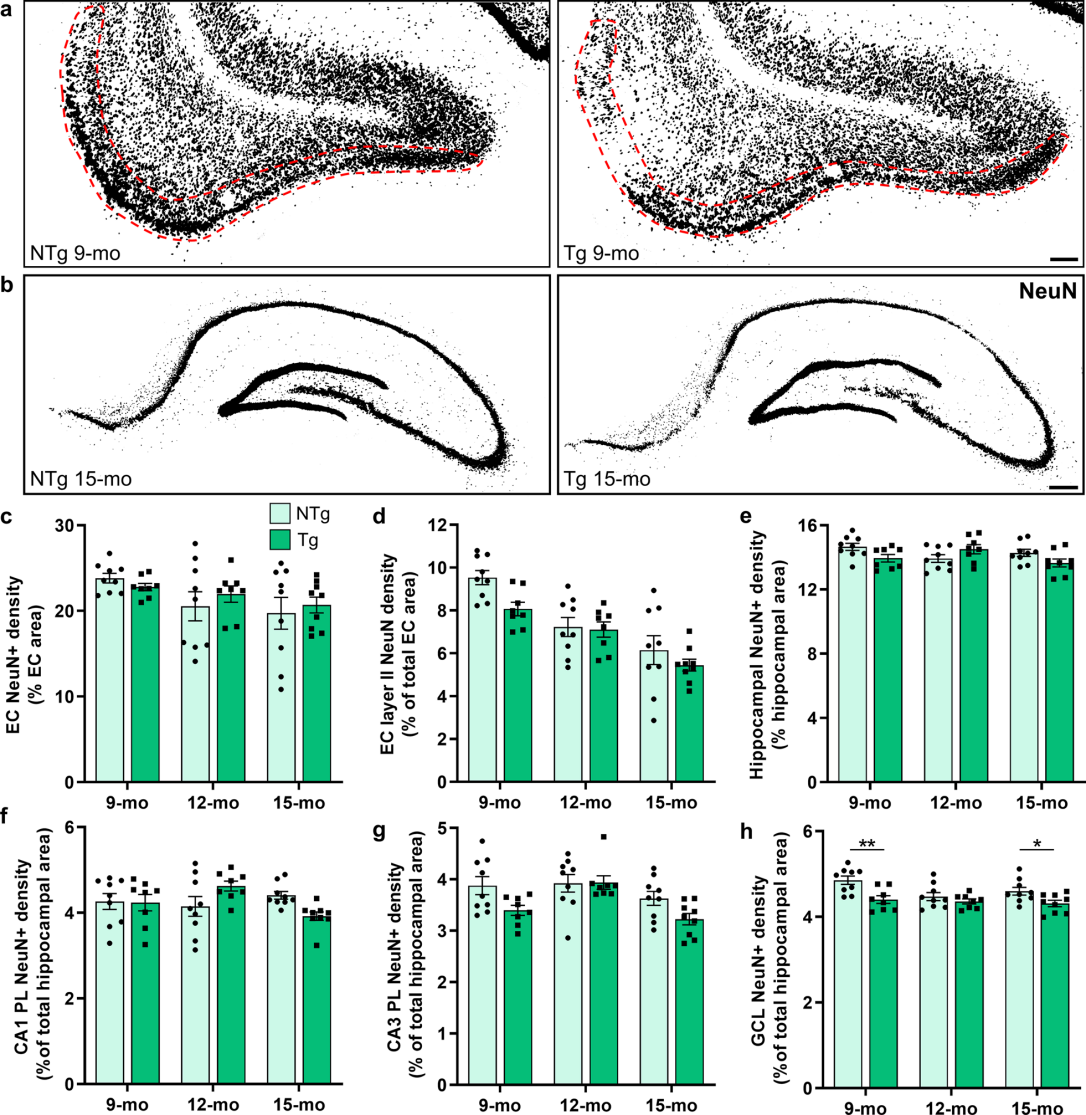
Early-stage neuronal loss precedes hippocampal neuronal compensation at 12 months, before robust decline. To assess neuronal loss in TgF344-AD rats, entorhinal cortical (EC) and hippocampal neurons were quantified by NeuN^+^ staining in NTg and TgF344-AD rats at 9, 12 and 15 months of age (*n* = 8–9 rats/genotype/age). **a** Representative NeuN^+^ staining in 9-month-old NTg and Tg rats demonstrates a loss of EC layer II neurons in TgF344-AD rats (red outline). **b** In the hippocampus, NeuN staining at 15 months demonstrates thinning of pyramidal and granular cell layers in Tg rats. **c** No significant genotype differences were detected at any age in the total EC neurons. **d** Tg rats exhibited a significant overall deficit (*P* = 0.03) in layer II EC neurons. **e, f** There were significant age × genotype interactive effects (both *P* = 0.02) on the total hippocampal neurons and the CA1 pyramidal layer (PL) neurons. The Tg rats exhibited an overall significant loss of **g** CA3 PL neurons (*P* = 0.02) and **h** DG granular cell layer (GCL) neurons (*P* = 0.0002), notably at 9 and 15 months. Scale bars, 200 μm (**a**) and 400 μm (**b**). Data are mean ± SEM; two-way ANOVA with correction for multiple comparisons with a Holm-Sidak post hoc test (see Table 1 for complete statistics); *P < 0.05, **P < 0.01

We hypothesized that staging the alterations of entorhinal cortical and hippocampal neurons may elucidate mechanisms present in AD that indicate higher resilience to neuronal dysfunction or loss [16, 17, 19]. Early neuronal damage and subsequent loss occurred in both the entorhinal cortex and hippocampus of TgF344-AD rats, with differing dynamics across age (Fig. 1). Representative images of 9-month-old NTg and TgF344-AD entorhinal cortices stained with NeuN, demonstrate a loss of NeuN immunoreactivity (NeuN^+^) in the layer II neurons of TgF344-AD (red outline; Fig. 1a). Representative images of hippocampal NeuN immunostaining in 15-month-old NTg and TgF344-AD rats demonstrate a loss of neuronal NeuN^+^, with significant thinning of pyramidal and granular cell layers (Fig. 1b). The decreased NeuN signal indicates either a downregulation of expression within neurons, indicative of neuronal injury, or neuronal death [32]. We quantified entorhinal cortical and hippocampal neurons by NeuN immunoreactivity across age and genotype, to elucidate the dynamics of neuronal damage in TgF344-AD rats. There was no genotype difference (*P* = 0.66), yet a small but significant age-related loss in the total NeuN^+^ entorhinal cortical neurons (*P* = 0.05; Fig. 1c; Table 1). We further quantified NeuN^+^ neurons in the layer II entorhinal cortical neurons, which are the major excitatory input into the dentate gyrus (DG) and are critical in hippocampal function [33, 34]. Both NTg and TgF344-AD rats consistently exhibited age-related loss of NeuN^+^ in layer II entorhinal cortical neurons (*P* < 0.0001), with significantly less neurons in TgF344-AD rats compared to NTg (overall genotype comparison across all ages: *P* = 0.03), suggesting that this age-related loss began earlier in TgF344-AD than in NTg rats. NTg-Tg multiple comparisons at 9 (*P* = 0.06), 12 (*P* = 0.84) and 15 (*P* = 0.42) months of age did not reveal significant differences (Fig. 1d; Table 1).

**Table 1:** Age and genotype statistics for entorhinal cortical and hippocampal neuronal changes

Readout	Age effect	Genotype effect	Age × genotype interaction	9-month: NTg *vs* Tg	12-month: NTg *vs* Tg	15-month: NTg *vs* Tg
EC NeuN (Fig. 1c)	*F*(2,46) = 3.29; *P = 0.05	*F*(1,46) = 0.20; *P* = 0.66	*F*(2,46) = 0.55; *P* = 0.58	N/A	N/A	N/A
EC layer II NeuN (Fig. 1d)	*F*(2,46) = 25.10; ***P < 0.0001	*F*(1,46) = 4.81; *P = 0.03	*F*(2,46) = 1.23; *P* = 0.30	*P* = 0.06	*P* = 0.84	*P* = 0.43
HP NeuN (Fig. 1e)	*F*(2,46) = 1.10; *P* = 0.34	*F*(1,46) = 1.65; *P* = 0.21	*F*(2,46) = 4.50; *P = 0.02	*P* = 0.13	*P* = 0.13	*P* = 0.13
HP CA1 NeuN (Fig. 1f)	*F*(2,46) = 0.96; *P* = 0.39	*F*(1,46) = 0.008; *P* = 0.93	*F*(2,46) = 4.48; *P = 0.02	*P* = 0.92	*P* = 0.10	*P* = 0.10
HP CA3 NeuN (Fig. 1g)	*F*(2,46) = 6.41; **P = 0.004	*F*(1,46) = 6.28; *P = 0.02	*F*(2,46) = 1.75; *P* = 0.19	*P* = 0.06	*P* = 0.94	*P* = 0.09
HP GCL NeuN (Fig. 1h)	*F*(2,46) = 3.35; *P = 0.04	*F*(1,46) = 16.30; ***P = 0.0002	*F*(2,46) = 1.87; *P* = 0.17	**P = 0.002	*P* = 0.36	*P = 0.04

Two-way ANOVA with correction for multiple comparisons (genotype differences per age) with a Holm-Sidak post hoc test

*EC* entorhinal cortex, *GCL* granular cell layer, *HP* hippocampus, *NeuN* neuronal nuclei, *NTg* non-transgenic, *Tg* transgenic

Significant effects are in bold text. *P < 0.05; **P < 0.01; ***P < 0.001

In the hippocampus, no significant overall age (*P* = 0.34) or genotype (*P* = 0.21) effects were detected on NeuN immunoreactivity, yet a significant age × genotype interaction was seen (*P* = 0.02; Fig. 1e; Table 1), indicating that the effect of genotype on hippocampal neuronal viability is dependent on and differs by age; and vice versa. Specifically, these data demonstrate a mid-stage compensation in the hippocampus of 12-month-old TgF344-AD rats, which contrasts with the progressive entorhinal cortical loss of neuronal NeuN^+^. We probed subregions of the hippocampus to better understand neuronal changes across genotype and age. In the CA1 pyramidal layer, no overall genotype (*P* = 0.93) or age (*P* = 0.39) effects were detected; however, similar to the total hippocampal NeuN^+^ immunoreactivity, a significant age × genotype interaction was detected (*P* = 0.02). This interactive effect highlights greater NeuN immunoreactivity in 12-month-old TgF344-AD rats, compared to 9- and 15-months (Fig. 1f; Table 1). In the CA3 pyramidal layer, there were significant effects of age (*P* = 0.004) and an overall TgF344-AD genotype loss of NeuN^+^ immunoreactivity (*P* = 0.02; Fig. 1g; Table 1). Similarly, significant age (*P* = 0.04) and genotype (*P* = 0.0002) effects were detected in the granular cell layer of the DG, with TgF344-AD loss of NeuN^+^ immunoreactivity at 9 and 15 months of age (*P* = 0.002 and *P* = 0.04, respectively), but not at 12 months of age (*P* = 0.36; Fig. 1h; Table 1). These data support a stage of neuronal compensation at 12 but not 9 or 15 months of age.

**Fig. 2:**
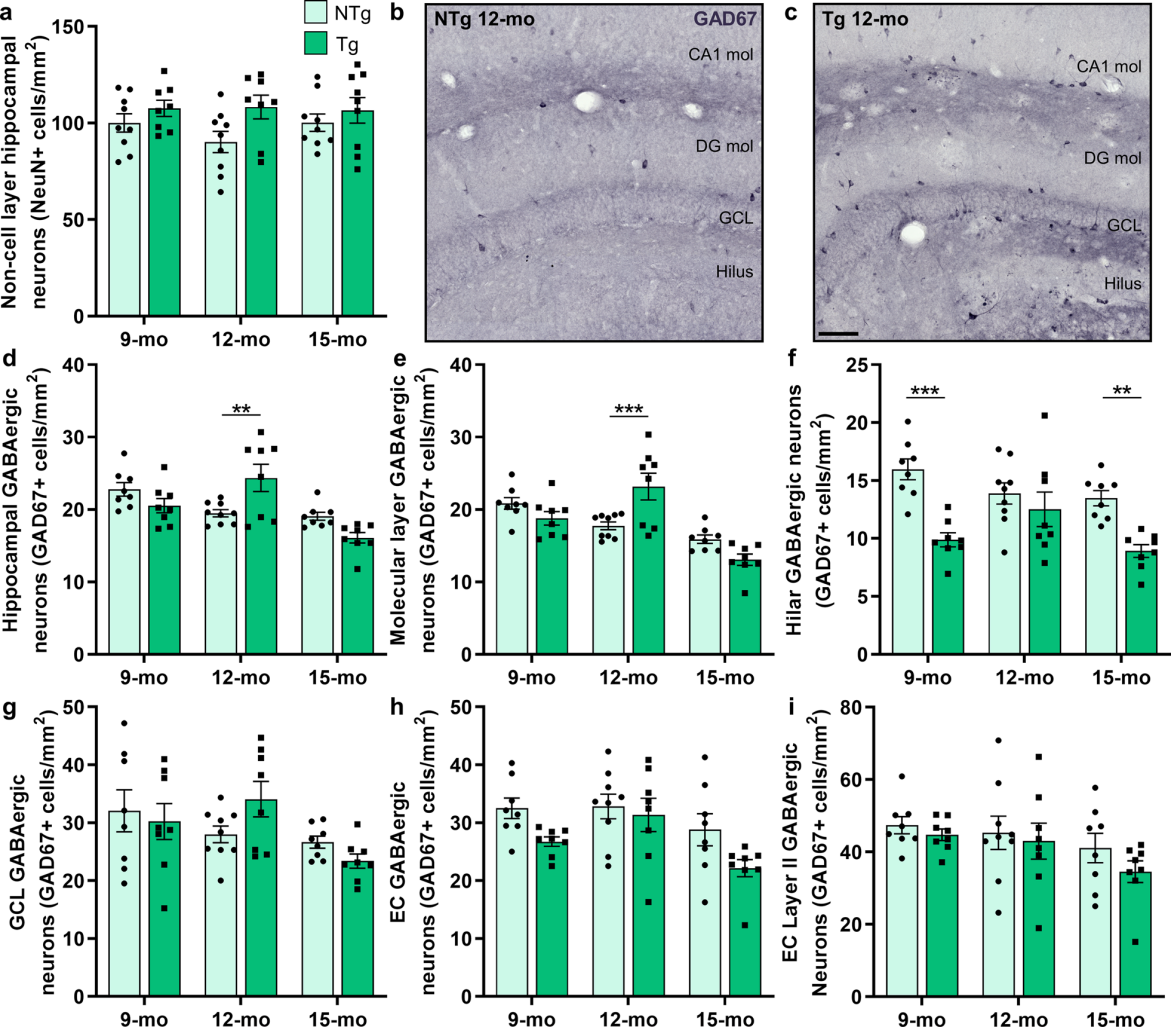
Resilience of GABAergic interneurons in TgF344-AD rats at 12 months of age. We probed neurons within the hippocampal molecular layers to determine if this population underlies the neuronal changes in TgF344-AD rats at 9, 12, and 15 months of age (*n* = 8–9 rats/genotype at each age). **a** Tg rats exhibited a significant overall genotype-related increase (*P* = 0.02) in non-cell layer NeuN^+^ neurons. To phenotype these cells, hippocampal GABAergic interneurons were assessed by GAD67 staining. **b** and **c** Representative hippocampal GAD67 staining in 12-month NTg and Tg rats demonstrates increased interneurons primarily in Tg hilus and molecular layers of CA1 and DG. Quantification of total hippocampal (**d**) and molecular-layer (**e**) GABAergic interneurons determined a significant increase in 12-month Tgs. **f** A large deficit was detected in hilar GABAergic interneurons in 9- and 15-month Tgs, but not at 12 months. **g** There were no significant genotype differences in GAD67 neurons in the granular cell layer (GCL). **h** Tg rats exhibited an overall significant loss of total EC GABAergic interneurons (*P* = 0.01) in the entorhinal cortex (EC), compared to NTgs. **i** Finally, there was no genotype-related loss of EC layer II GABAergic interneurons. Scale bar, 100 μm. Data are mean ± SEM; two-way ANOVA with correction for multiple comparisons with a Holm-Sidak post hoc test (see Table 2 for complete statistics); **P < 0.01, ***P < 0.001

The results of neuronal NeuN immunoreactivity within the pyramidal and granular cell layers indicate neuronal injury in 9-month-old TgF344-AD rats, and neuronal death at 15 months of age; however, we postulated that the neuronal compensation at 12 months is not fully represented within these subregions (Fig. 2). We thus investigated total neurons in the non-cell layers within the hippocampus and determined a significant increase of neurons immunoreactive for NeuN in TgF344-AD rats compared to NTg (genotype effect: *P* = 0.02); whereas no age or age × genotype effects were detected (*P* = 0.65, *P* = 0.49, respectively; Fig. 2a; Table 2).

**Table 2:** Age and genotype statistics for entorhinal cortical and hippocampal inhibitory interneuron changes

Readout	Age effect	Genotype effect	AgeXgenotype interaction	9-month: NTg-Tg	12-month: NTg-Tg	15-month: NTg-Tg
HP non-cell layer NeuN (Fig. 2a)	*F*(2,46) = 0.44; *P* = 0.65	*F*(1,46) = 5.90; *P = 0.02	*F*(2,46) = 0.72; *P* = 0.49	*P* = 0.55	*P* = 0.07	*P* = 0.55
HP GAD67 (Fig. 2d)	*F*(2,43) = 11.35; ***P = 0.0001	*F*(1,43) = 0.02; *P* = 0.89	*F*(2,43) = 9.22; ***P = 0.0005	*P* = 0.13	**P = 0.004	*P* = 0.10
HP molecular layer GAD67 (Fig. 2e)	*F*(2,43) = 21.35; ***P < 0.0001	*F*(1,43) = 0.04; *P* = 0.84	*F*(2,43) = 10.55; ***P = 0.0002	*P* = 0.15	***P = 0.001	*P* = 0.11
HP hilus GAD67 (Fig. 2f)	*F*(2,43) = 2.83; *P* = 0.07	*F*(1,43) = 28.97; ***P < 0.0001	*F*(2,43) = 3.55; *P = 0.04	***P < 0.0001	*P* = 0.29	**P = 0.002
HP GCL GAD67 (Fig. 2g)	*F*(2,43) = 4.07; *P = 0.02	*F*(1,43) = 0.03; *P* = 0.87	*F*(2,43) = 2.15; *P* = 0.13	N/A	N/A	N/A
EC GAD67 (Fig. 2h)	*F*(2,43) = 5.05; **P = 0.01	*F*(1,43) = 7.23; **P = 0.01	*F*(2,43) = 0.88; *P* = 0.42	*P* = 0.12	*P* = 0.62	*P* = 0.10
EC layer II GAD67 (Fig. 2i)	*F*(2,43) = 2.69; *P* = 0.08	*F*(1,43) = 1.60; *P* = 0.21	*F*(2,43) = 0.20; *P* = 0.82	N/A	N/A	N/A

Two-way ANOVA with correction for multiple comparisons (genotype differences per age) with a Holm-Sidak post hoc test

*EC* entorhinal cortex, *GAD67* glutamate decarboxylase 67, *GCL* granular cell layer, *HP* hippocampus, *NeuN* neuronal nuclei, *NTg* non-transgenic, *Tg* transgenic

Significant effects are in bold text. *P < 0.05; **P < 0.01; ***P < 0.001

Based on their localization in molecular and polymorph layers and the role of inhibitory tone in regulating hyperexcitability and cognition in AD [13, 14], we hypothesized that GABAergic interneurons may contribute to resilience in 12-month-old TgF344-AD rats. GABAergic alterations have been well-documented in AD (reviewed in [13, 14]), including reports of increased GABAergic neurons, synapses and activity [35, 36]. We utilized the GABAergic interneuron marker GAD67 to assess hippocampal inhibitory neurons across age and genotypes. GAD67 immunostaining in 12-month-old NTg (Fig. 2b) and TgF344-AD rats (Fig. 2c) demonstrated a noticeable increase in the population of GAD67 immunoreactive (GAD67^+^) interneurons in TgF344-AD rats. We quantified hippocampal GAD67^+^ interneurons and determined no overall genotype effect (*P* = 0.89), yet a significant effect of age (*P* = 0.0001) and age × genotype interaction (*P* = 0.0005), representing mid-stage compensation in TgF344-AD rats. The TgF344-AD rats at 12 months exhibited significantly more hippocampal GAD67^+^ neurons compared to NTg (*P* = 0.004), with no difference at 9 months (*P* = 0.13) or 15 months of age (*P* = 0.10) **(**Fig. 2d; Table 2).

Next, we assessed whether the GABAergic interneuron population accounts for the changes in the total hippocampal neurons in TgF344-AD rats, by assessing subregional distribution of GAD67^+^ cells, in relation to NeuN^+^ cell changes. Genotype differences in GAD67^+^ cells were primarily observed within the CA1, DG and hilus, while less prominent in the granular cell layer. The TgF344-AD hippocampal molecular layers similarly exhibited no overall genotype effect (*P* = 0.84), with a significant loss over age (*P* < 0.0001) and an age × genotype interaction (*P* = 0.0002); notably, a recovery of GAD67 immunoreactivity in 12-month-old TgF344-AD rats was observed, compared to NTg (*P* = 0.001; Fig. 2e; Table 2). The most robust loss of GAD67 immunoreactive neurons occurred in the hilus of TgF344-AD rats compared to age-matched NTg rats (genotype: *P* < 0.0001; age: *P* = 0.07; interaction: *P* = 0.04), with a significant decrease at 9 and 15 months of age (*P* < 0.0001 and *P* = 0.002, respectively), whereas no difference was detected at 12 months of age (*P* = 0.29; Fig. 2f; Table 2). There were no significant differences in GAD67-immunoreactive GABAergic interneurons in the GCL (Fig. 2g; Table 2), or in the pyramidal layers of the CA1 or CA3 (Additional file 1: Fig. S1), between the genotypes. These data indicate that the loss of inhibitory neuronal GAD67 immunoreactivity in 9- and 15-month-old TgF344-AD rats occurs primarily within the hilus. We observed decreased NeuN-immunoreactive cells primarily in hippocampal granular cell layer, and CA1 and CA3 pyramidal layers of TgF344-AD rats, whereas GAD67-immunoreactive inhibitory interneurons remained relatively unchanged in these cell layers. Therefore, the GABAergic cell population does not correspond to the loss of NeuN immunoreactivity observed within hippocampal cell layers in TgF344-AD rats, thus indicative of injury of primarily excitatory granular and pyramidal neurons. Conversely, neuronal resilience in 12-month-old TgF344-AD rats consists of GABAergic interneurons, primarily within the hippocampal molecular layers.

The entorhinal cortical GABAergic interneurons exhibited an overall loss of GAD67 immunoreactivity over age and in TgF344-AD rats (both *P* = 0.01) compared to NTg rats (Fig. 2h; Table 2). Interestingly, no significant genotype differences in GABAergic interneurons were detected in layer II of the entorhinal cortex (*P* = 0.21; Fig. 2i; Table 2), suggesting that the neuronal injury at 9 months of age within this region was primarily within DG-projecting excitatory neurons [33, 34]. In light of the mid-stage GABAergic neuronal changes, it is possible that this early impairment in hippocampal-projecting neurons in the entorhinal cortex of TgF344-AD rats may be the trigger for GABAergic remodeling in the DG [37].

### Somatostatin neurodegeneration yet parvalbumin resistance to early-stage tau accumulation

**Fig. 3:**
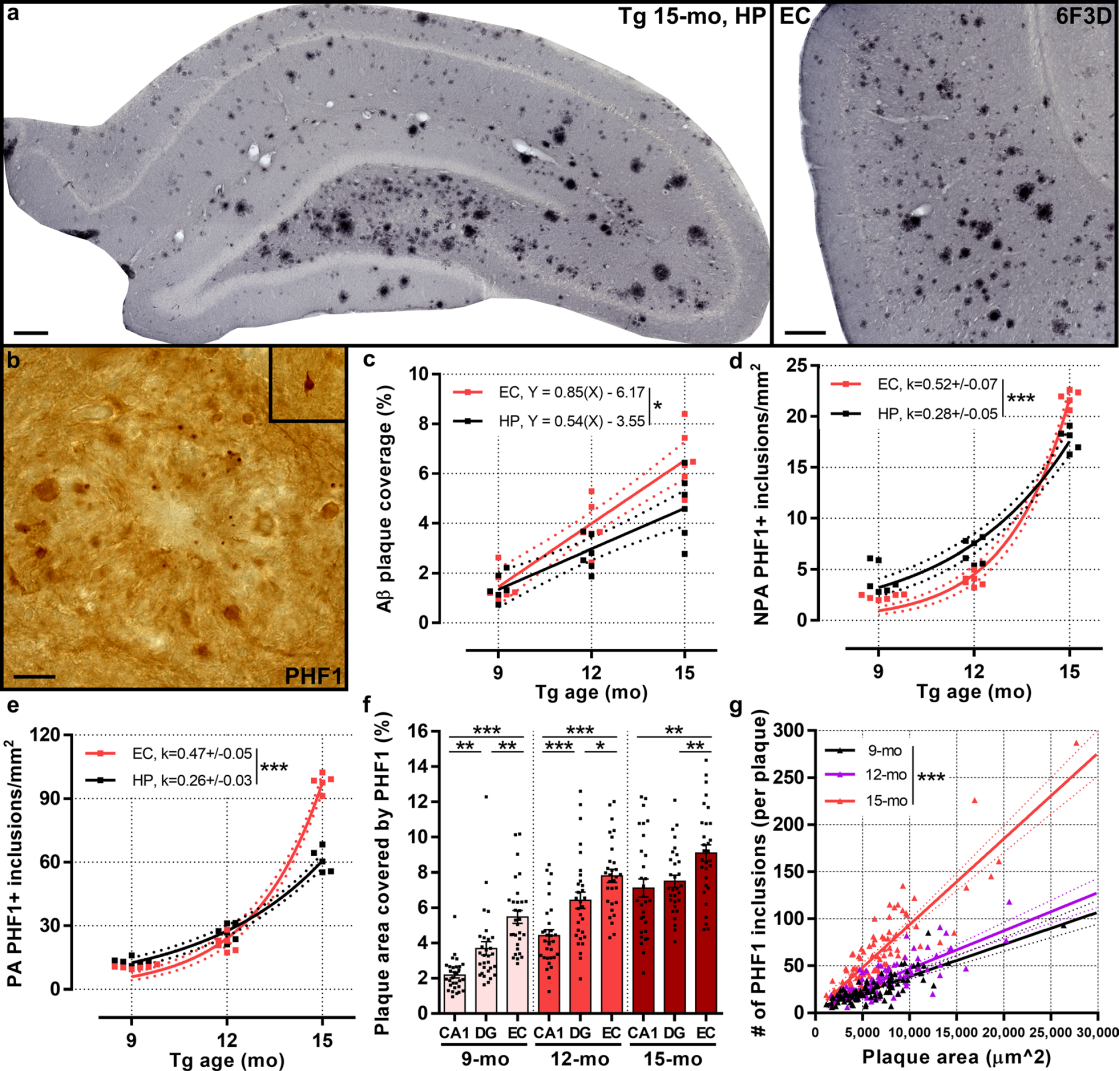
Linear Aβ deposition and exponential tau accumulation across disease progression in TgF344-AD rats. Aβ and tau pathology were assessed in the hippocampus (HP) and entorhinal cortex (EC) of 9-, 12- and 15-month-old TgF344-AD rats (*n* = 5–6 rats/age). **a** Representative HP and EC images of 6F3D staining for Aβ demonstrate extensive plaque coverage at 15 months of age. **b** PHF1 staining indicates dystrophic neurites [plaque-associated (PA)] and tangle inclusions [non-plaque-associated (NPA); inset] of hyperphosphorylated tau. **c** Quantification of plaque coverage in HP and EC demonstrates linear accumulation of Aβ across age. NPA (**d**) and PA (**e**) tau inclusions also increased with age but in an exponential manner, notably between 12 and 15 months of age, and to a significantly greater degree in EC than HP. **f** Quantification of PA inclusions per plaque in CA1, DG and EC determined significant effects of age and region on plaque area covered by tau dystrophic neurites (*n* = 30 plaques/region in each cohort of 5 rats/age). Significantly greater plaque area was covered by tau inclusions in EC compared to DG and CA1 at all ages, and DG compared to CA1 at 9 and 12 months. **g** Linear relationship between plaque size and number of tau-positive dystrophic neurites, demonstrating significantly more neurites per plaque with age, most prominently at 15 months (9-month: Y = 0.003 X + 4.52; 12-month: Y = 0.004 X + 6.15; 15-month: Y = 0.009 X + 2.57; *n* = 90 plaques in each cohort of 5 rats/age). Scale bars, 200 μm (**a**) and 20 μm (**b**). Data are mean ± SEM (**f**) or 95% confidence intervals (**c–e, g**); two-way ANOVA with Holm-Sidak post hoc test (**f**), linear regression (**c, g**), non-linear exponential growth regression (**d, e**); *P < 0.05, **P < 0.01, ***P < 0.001

To determine the relationship between disease progression and GABAergic neurons in TgF344-AD rats, we characterized hippocampal and entorhinal cortical Aβ and tau pathology across age. Aβ images in the 15-month-old TgF344-AD hippocampus and entorhinal cortex demonstrate significant amyloid plaques (Fig. 3a). PHF1^+^ inclusions demonstrated dystrophic neurite (Fig. 3b) and pre-tangle (Fig. 3b inset) pathology in TgF344-AD rats. We quantified hippocampal and entorhinal cortical areas (see Additional file 1: Fig. S2 for area selections) covered by Aβ plaques stained with 6F3D and detected a significant effect of age [*F*(2,15) = 18.58, *P* < 0.0001 and *F* (2,15) = 41.11, *P* < 0.0001, respectively], with more plaque coverage at 12- compared to 9-month age (*P* = 0.02 and *P* = 0.0007, respectively), and at 15- compared to 12-month age (*P* = 0.006 and *P* = 0.0005, respectively). Aβ accumulated linearly across age, at a significantly greater rate in the entorhinal cortex (Y = 0.85 X-6.17) compared to the hippocampus [Y = 0.54 X−3.55; *F*(1,32) = 5.76, *P* = 0.02; Fig. 3c].

TgF344-AD rats exhibit hyperphosphorylated tau inclusions consisting of endogenous rat tau [20, 21, 24]. We have previously described the presence of both non-plaque-associated tangle-like tau inclusions and plaque-associated dystrophic neurite tau inclusions in TgF344-AD rats [24]. Here, we quantified these inclusions in the entorhinal cortex and hippocampus of TgF344-AD rats (see Additional file 1: Fig. S2 for area selections) at 9, 12 and 15 months. In the entorhinal cortex and the hippocampus, there was a significant effect of age on the number of non-plaque-associated inclusions [*F*(2,14) = 1882, *P* < 0.0001 and *F*(2,14) = 165.9, *P* < 0.0001, respectively] and plaque-associated inclusions [*F*(2,14) = 1162, *P* < 0.0001 and *F*(2,14) = 202.0, *P* < 0.0001, respectively]. In contrast to the linear accumulation of Aβ plaques, tau inclusions spread exponentially with age. The exponential growth of tau pathology in the entorhinal cortex was significantly greater than that in the hippocampus for non-plaque-associated inclusions [entorhinal cortex: k = 0.52 ± 0.07, hippocampus: k = 0.28 ± 0.05; *F*(1,30) = 38.01, *P* < 0.0001; Fig. 3d] and plaque-associated inclusions [entorhinal cortex: k = 0.47 ± 0.05, hippocampus: k = 0.26 ± 0.03; *F*(1,30) = 50.34, *P* < 0.0001; Fig. 3e]. A significant effect of region [*F*(2,261) = 40.85, *P* < 0.0001] and age [*F*(2,261) = 83.94, *P* < 0.0001] was detected on the plaque area covered by tau inclusions, with the entorhinal cortex exhibiting significantly greater coverage than hippocampal regions (DG and CA1), at all ages (Fig. 3f). Finally, there were significantly more tau inclusions per plaque with increased plaque size and age in TgF344-AD rats (9-month Y = 0.003 X + 4.52, 12-month Y = 0.004 X + 6.15, 15-month Y = 0.009 X + 2.57; with significant differences in slope, *F*(2,264) = 63.23, *P* < 0.0001, Fig. 3g). This indicates that the tau pathology is not linked to the linear increase in Aβ pathology, and recapitulates Braak staging [25] with higher rates of accumulation in the entorhinal cortex compared to hippocampus.

**Fig. 4:**
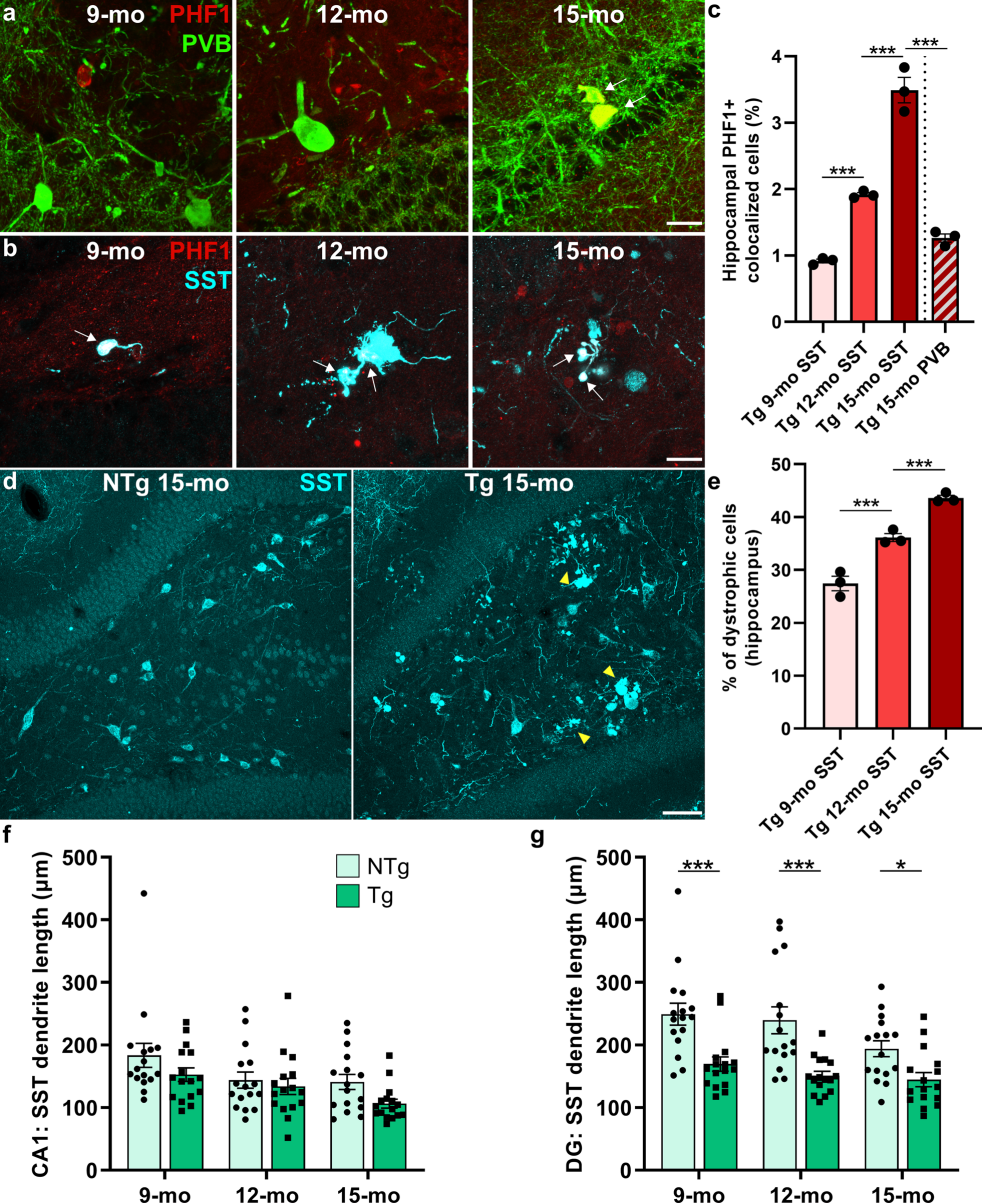
Early-stage tau and Aβ disrupt hippocampal somatostatin interneurons, while parvalbumin cells are spared until 15 months. We assessed the accumulation of tau pathology within hippocampal interneurons of 9-, 12- and 15-month-old TgF344-AD rats, by co-localization of PHF1 with GABAergic subtypes: PVB and SST (representative images from *n* = 6–12 sections/group). **a** No co-localization was detected between PHF1 and PVB at 9 and 12 months of age. At 15 months, hippocampal PVB neurons exhibited cytoplasmic tau accumulation (white arrows). **b** Conversely, hippocampal SST interneurons exhibited cytoplasmic (white arrows), and less commonly dendritic PHF1 staining at as early as 9 months of age. **c** Quantification of SST^+^/PHF1^+^ co-localization at 9, 12 and 15 months of age, and PVB^+^/PHF1^+^ co-localization at 15 months in Tg rats showed significant increases in SST-tau pathology over age, and more affected cells than PVB-tau at 15 months. **d** Compared to NTg, the 15-month-old Tg rats had a visually degenerative hippocampal SST network with stunted processes, and cell bodies that clustered around Aβ plaques (yellow arrowheads). **e** The percentage of dystrophic SST neurons (quantified as plaque-associated) significantly increases with age in Tg rats. **f** Quantification of dendritic length from SST neuronal traces showed overall significant genotype differences in CA1 (*P* = 0.02). **g** In the DG, Tg rats exhibited a significant loss in SST dendritic length at all 3 ages. Scale bars, 20 μm (**a, b**) and 100 μm (**c**). Data are mean ± SEM (**c, e:** *n* = 3 rats/age, 6 hippocampi/rat**; f, g:** from *n* = 16 cells/cohort, sampled across 4 rats/genotype at each age); one-way (**c** and **e**) and two-way (**f** and **g**) ANOVA with correction for multiple comparisons with a Holm-Sidak post hoc test; *P < 0.05; ***P < 0.001

To delineate the effect of tau pathology on neuronal deficits and resilience, we examined whether hyperphosphorylated tau accumulates within inhibitory and/or excitatory neurons in TgF344-AD rats. We utilized 6–12 sections (both hemispheres) per age per marker across multiple rats, with an exhaustive search through the entire brain region including the z plane (Fig. 4 and Additional file 1: Fig. S3). PHF1 did not co-localize with excitatory neuronal markers at any age in either the entorhinal cortex or the hippocampus (Additional file 1: Fig. S3), suggesting that the excitatory neuronal loss in TgF344-AD rats is driven by Aβ pathology; alternatively, we cannot rule out the possibility that tau pathology caused excitatory neuronal death prior to detection of the PHF1 epitope. We next examined PVB- and SST-expressing inhibitory interneurons for tau co-localization, as their hippocampal populations exhibit distinct contributions to cognitive function and AD pathogenesis [6, 38–41]. No tau inclusions were detected within hippocampal PVB^+^ neurons in 9- and 12-month-old TgF344-AD rats, whereas at 15 months of age, cytoplasmic tau inclusions were detected within PVB^+^ interneurons (Fig. 4a). The PHF1^+^/PVB^+^ interneurons typically exhibited stunted processes in comparison to those without tau aggregates, indicative of a progression towards degeneration. Interestingly, the hippocampal SST^+^ interneurons exhibited PHF1 co-localization at all three ages in TgF344-AD rats, although rare at 9 months in comparison to 12 and 15 months of age (Fig. 4b). Tau accumulated predominantly in the cytoplasm, and was less observed in processes of SST^+^ neurons (Additional file 1: Fig. S3), whereas the PVB^+^ neurons had tau inclusions exclusively in the cytoplasm. In the entorhinal cortex, PHF1^+^/PVB^+^ cells were detectable at all ages, while SST^+^ interneurons were negative for PHF1^+^ staining. The entorhinal cortical SST^+^ interneurons displayed degenerative morphology, and surrounded plaques (Additional file 1: Fig. S3f). We quantified hippocampal PHF1^+^/SST^+^ neurons at 9, 12 and 15 months of age, and PHF1^+^/PVB^+^ neurons at 15-months in TgF344-AD rats, and found significant increases of PHF1^+^/SST^+^ neurons over age, and compared to PHF1^+^/PVB^+^ neurons at 15 months [overall ANOVA: *F*(3,8) = 122.0; *P* < 0.0001; Fig. 4c]. These combined data indicate that the inhibitory but not excitatory neurons develop intracellular tau inclusions in TgF344-AD rats, and degeneration of SST-expressing neurons may precede PVB neurodegeneration in the hippocampus.

Representative images of hippocampal SST^+^ interneuronal staining at 15 months of age demonstrated a degenerative SST neuronal network in TgF344-AD rats, including a loss of dendritic organization and clustering of SST^+^ cells around plaques (Fig. 4d). This was consistent with 9 and 12 months of age, but to a lesser degree. To determine the degree of dystrophy in the hippocampal SST network, SST^+^ interneurons were quantified as plaque-associated and non-plaque-associated in 9-, 12- and 15-month-old TgF344-AD rats. Results showed significant increases over age [*F*(2,6) = 72.61; *P* < 0.0001], and the dystrophic SST neurons made up > 40% of the SST hippocampal population at 15 months of age (Fig. 4e).

To further characterize hippocampal SST^+^ neuronal atrophy, tracing of processes was conducted to examine changes in dendritic arborization. In the CA1 region of the hippocampus, there was a significant effect of age [*F*(2,90) = 5.96; *P* = 0.004] and genotype [*F*(1,90) = 5.60; *P* = 0.02] on SST^+^ dendritic length, with no significant age × genotype interaction [*F*(2,90) = 0.52; *P* = 0.60], representing overall shorter processes in TgF344-AD rats (Fig. 4f). In the DG, there were significant effects of age [*F*(2,90) = 3.98; *P* = 0.02] and genotype [*F*(1,90) = 38.27; *P* < 0.0001], but no significant age × genotype interaction [*F*(2,90) = 1.04; *P* = 0.36], and TgF344-AD rats exhibited significantly shorter SST^+^ processes at 9 (*P* = 0.0003), 12 (*P* = 0.0001) and 15 months of age (*P* = 0.02; Fig. 4g). Overall, the early and progressive degeneration of hippocampal SST^+^ interneurons was impacted by both tau and Aβ pathology.

### Neuroplastic reorganization of the hippocampal parvalbumin neuronal network

**Fig. 5:**
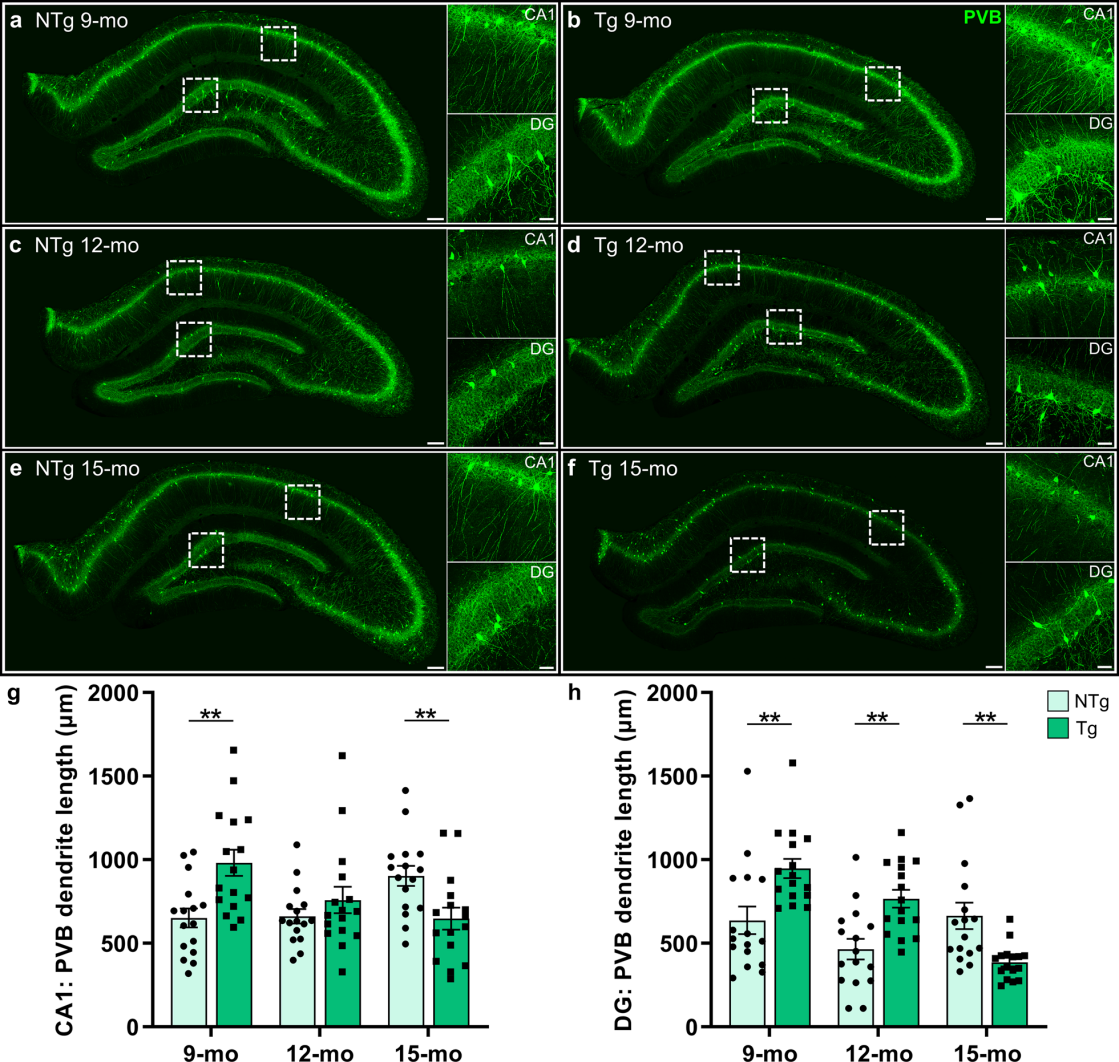
Compensatory hippocampal parvalbumin remodeling in TgF344-AD rats. We hypothesized that the hippocampal PVB network would undergo neuroplastic reorganization in TgF344-AD rats, coinciding with GABAergic compensation. We assessed PVB (green) staining in 9-, 12- and 15-month-old NTg and Tg rats (representative images from *n* = 6 rats/genotype at each age). At 9 months, compared to NTgs (**a**), Tg (**b**) rats exhibited increased PVB staining, with greater complexity of processes. **c**, **d** At 12 months of age, the overall PVB staining density was decreased; however, the Tg (**d**) rats exhibited increased complexity in the DG. **e**, **f** At 15 months of age, there was a robust loss of PVB staining density and complexity in Tg rats throughout the hippocampus, compared to NTg rats and compared to younger ages. **g** Quantification of dendritic length from CA1 PVB neuronal traces showed significantly longer dendrites in Tg rats at 9 months, no differences at 12 months, and a loss at 15 months of age, compared to NTgs. **h** In the DG, Tg PVB neurons exhibited significantly increased dendritic length at 9 and 12 months of age and reduced dendritic length at 15 months compared to NTgs. Scale bars, 200 μm (whole hippocampus) and 50 μm (CA1/DG). Data are mean ± SEM (from *n* = 16 cells/cohort, sampled across 4 rats/genotype at each age); two-way ANOVA with correction for multiple comparisons with a Holm-Sidak post hoc test; **P < 0.01

We hypothesized that the hippocampal PVB^+^ GABAergic neuronal network would exhibit neuroplasticity in TgF344-AD rats as a compensation for SST impairments, coinciding with resilience to tau pathology. We utilized PVB labelling of GABAergic neurons to visualize these processes (Fig. 5). The PVB^+^ dendritic processes spread throughout the hippocampal subregions and molecular layers, with a high complexity of primary and secondary projections from PVB^+^ interneurons, and dense staining throughout the pyramidal and granular cell layers. Interestingly, at 9 and 12 months, the TgF344-AD rats exhibited increased PVB staining and processes in the DG and CA1 (Fig. 5a-d), yet at 15 months the TgF344-AD rats exhibited a loss throughout the hippocampus (Fig. 5e, f), compared to the age-matched NTg rats.

We traced PVB^+^ neuronal processes to examine potential genotype differences in dendritic length. In the CA1, there were no overall effects of age [*F*(2,90) = 1.34; *P* = 0.27] and genotype [*F*(1,90) = 1.16; *P* = 0.29], with a significant age × genotype interaction effect on dendritic length [*F*(2,90) = 10.22; *P* < 0.0001]. The TgF344-AD rats at 9 months of age exhibited significantly longer PVB^+^ dendrites than NTgs (*P* = 0.002), with no differences at 12 months (*P* = 0.30) and a significantly shorter dendritic length at 15 months compared to NTgs (*P* = 0.01; Fig. 5g). In the DG, significant overall effects of age [*F*(2,90) = 9.34; *P* = 0.0002], genotype [*F*(1,90) = 4.76; *P* = 0.03] and their interaction [*F*(2,90) = 14.37; *P* < 0.0001] were detected on PVB^+^ dendritic length, with TgF344-AD increases at 9 and 12 months (both *P* = 0.002), and a decrease at 15 months (*P* = 0.003), compared to NTgs (Fig. 5h). These data demonstrated significant inhibitory remodeling in TgF344-AD rats, prior to atrophy of PVB^+^ inhibitory interneurons at 15 months of age.

**Fig. 6:**
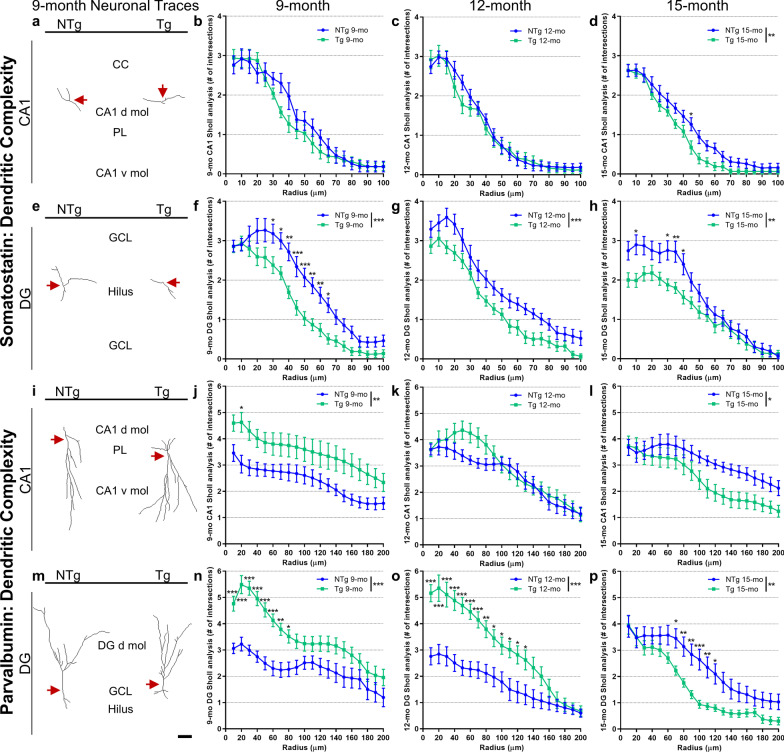
Dense parvalbumin dendritic connective zone in TgF344-AD rats in contrast with atrophic somatostatin interneurons. We further probed the hippocampal GABAergic network in TgF344-AD rats through neuronal tracing with Sholl analyses to determine the dendritic complexity (branching intersections by distance from soma). SST and PVB neurons were assessed in CA1 and DG in NTgs and Tgs at 9, 12 and 15 months of age. No significant differences were detected in CA1 SST dendritic complexity at 9 (**a, b**) and 12 months (**c**), but there was a loss of complexity in 15-month-old Tgs (**d**). **e**–**h** In the DG, Tg SST interneurons had decreased intersections at all ages, indicative of vast neuronal atrophy. **i**–**l** For CA1 PVB interneurons, increased dendritic complexity was detected in Tgs at 9 months (**i** and **j**) and 12 months (significant genotype × distance interaction: *P* = 0.03; **k**), but the dendritic complexity was decreased compared to NTgs at 15 months (**l**)**.** Notably, the DG PVB neurons of Tg rats exhibited a large increase in dendritic complexity near cell somas at 9 (**m** and **n**) and 12 months (**o**), and a robust loss of complexity at 15 months (**p**), compared to NTgs. **a, e, i, **and **m** Representative neuronal traces at 9 months demonstrate no change in CA1 SST, decreased DG SST dendritic length and complexity in Tgs, and increased PVB dendritic length and complexity in the Tg CA1 and DG. Red arrows indicate cell soma. Scale bar, 50 μm. Abbreviations: corpus callosum (cc); dorsal/ventral molecular layer (d/v mol); granular cell layer (GCL); pyramidal layer (PL). Data are mean ± SEM (from *n* = 16 cells/cohort, sampled across 4 rats/genotype at each age); repeated measures ANOVA with Holm-Sidak post hoc test; *P < 0.05, **P < 0.01, ***P < 0.001

To further probe inhibitory hippocampal remodeling and degeneration, we assessed the complexity of SST^+^ and PVB^+^ dendritic branching, as another measure of neuronal health. Sholl analyses were conducted on CA1 and DG neurons in TgF344-AD and NTg rats (Fig. 6). Specifically, branch points in SST^+^ and PVB^+^ projections, defined as dendritic intersections, were quantified as a function of distance from the cell soma. The CA1 SST^+^ neurons were not significantly different between NTg and TgF344-AD rats at 9 [genotype: *F*(1,30) = 1.42, *P* = 0.24; genotype × distance interaction: *F*(19,570) = 1.15, *P* = 0.30; Fig. 6a, b] and 12 months of age [genotype: *F*(1,30) = 0.33, *P* = 0.57; genotype × distance interaction: *F*(19,570) = 0.73, *P* = 0.79; Fig. 6c]. At 15 months of age, the TgF344-AD rats exhibited a small, yet significant, decrease in SST^+^ dendritic complexity [genotype: *F*(1,30) = 6.84, *P* = 0.01; genotype × distance interaction: *F*(19,570) = 1.11, *P* = 0.34; Fig. 6d]. In the DG, the TgF344-AD SST^+^ interneurons demonstrated atrophy at 9 [genotype: *F*(1,30) = 32.90, *P* < 0.0001; genotype × distance interaction: *F*(19,570) = 1.91, *P* = 0.01; Fig. 6e, f], 12 [genotype: *F*(1,30) = 13.18, *P* = 0.001; genotype × distance interaction: *F*(19,570) = 0.36, *P* = 0.99; Fig. 6g], and 15 months of age [genotype: *F*(1,30) = 10.57, *P* = 0.003; genotype × distance interaction: *F*(19,570) = 2.06, *P* = 0.005; Fig. 6h]. Representative neuronal traces demonstrated no differences in CA1 SST^+^ cells (Fig. 6a), and a loss of complexity in DG SST^+^ cells in 9-month-old TgF344-AD rats (Fig. 6e) in comparison to NTg neuronal traces. Consistent with the observations above (Fig. 4), SST^+^ neurons in TgF344-AD rats exhibited progressive region-specific neurodegeneration starting as early as 9 months of age.

In contrast, the TgF344-AD rats exhibited increased PVB^+^ complexity with a significantly greater number of PVB^+^ dendritic intersections in the CA1 region at 9 months of age [*F*(1,30) = 8.57, *P* = 0.007; no genotype × distance effect: *F*(19,570) = 0.30, *P* = 0.99; Fig. 6i, j]; which slightly decreased at 12 months of age in CA1, but with slight increases at 40–80 μm from the soma in TgF344-AD [no genotype differences: *F*(1,30) = 0.83, *P* = 0.37; significant genotype × distance interaction: *F*(19,570) = 1.76, *P* = 0.03; Fig. 6k]. Finally, at 15 months of age, the TgF344-AD rats had significant genotype differences in CA1 [*F*(1,30) = 6.03, *P* = 0.02; genotype × distance interaction: *F*(19,570) = 1.30, *P* = 0.18], representing a slight loss of complexity in TgF344-AD rats far (> 110 μm) from the CA1 somas (Fig. 6l).

Notably, in the DG of 9- and 12-month-old TgF344-AD rats, there was a ~ 1.5–2-fold significant increase in the number of PVB^+^ intersections within 80 μm from the cell soma, compared to NTg, indicative of a complex dendritic connective zone [significant effect of genotype: *F*(1,30) = 28.31, *P* < 0.0001 and *F*(1,30) = 17.66, *P* = 0.0002, respectively; genotype × distance interaction: *F*(19,570) = 2.77, *P* < 0.0001 and *F*(19,570) = 6.43, *P* < 0.0001, respectively; Fig. 6m-o]. Robust loss of PVB^+^ complexity occurred in the 15-month-old TgF344-AD DG compared to NTgs [significant effect of genotype: *F*(1,30) = 10.82, *P* = 0.003; and of genotype × distance interaction: *F*(19,570) = 2.28, *P* = 0.002; Fig. 6p]. Representative CA1 and DG PVB neuronal traces demonstrated increased dendritic intersections near cell soma in TgF344-AD rats at earlier ages that preceded atrophy later at 15 months of age (Fig. 6i, m; Additional file 1: Fig. S4). No genotype differences were observed in PVB^+^ and SST^+^ neurons at 4 months (Additional file 1: Fig. S5), a pre-pathology stage in TgF344-AD rats [42], indicating that the interneuron networks developed normally in TgF344-AD rats. The results further support that the compensatory and degenerative changes in these neuronal populations in 9-, 12- and 15-month-old TgF344-AD rats are linked to mounting Aβ and tau pathologies.

### TgF344-AD rats exhibit cognitive reserve in executive function

**Fig. 7:**
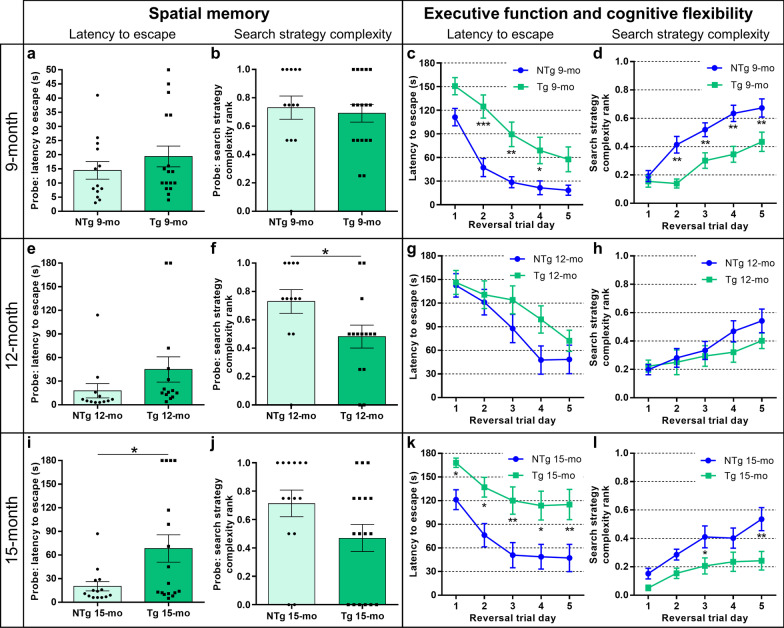
TgF344-AD rats exhibit cognitive resilience at 12 months of age and cognitive loss at 9 and 15 months. NTg and TgF344-AD rats were tested for spatial learning, memory and executive function in the Barnes maze at 9 (*n* = 13 and 17), 12 (*n* = 12 and 14) and 15 (*n* = 14 and 17) months of age. The latency to escape and the complexity of search strategies utilized were assessed in spatial memory probe test, and during 5 days of reversal learning. At 9 months, no differences were detected in the spatial memory latency to escape (**a**) or the search strategy complexity (**b**). Tg rats exhibited impaired executive function at 9 months, with significantly longer latency to escape (**c**) and less complex strategies (**d**) in reversal trials. The 12-month-old Tg rats had impaired spatial memory, with no deficits in the latency to escape (**e**), but significant deficits in search strategy complexity (**f**). **g** and **h** Despite prior deficits, the 12-month-old Tg rats exhibited cognitive maintenance in executive function, and cognitive flexibility, with no significant genotype effects. At 15 months of age, significant Tg deficits were detected in spatial memory latency to escape (**i**), but not search strategy complexity (**j**). (**k** and **l**) Robust deficits were detected in executive function and cognitive flexibility at 15 months. Data are mean ± SEM; unpaired *t*-test (**a, e**, and **i**), Mann–Whitney U test (**b, f, **and **j**), repeated measures ANOVA with Holm-Sidak post hoc test (**c, d, g, h, k**, and **l**); *P < 0.05, **P < 0.01, ***P < 0.001

We utilized the Barnes maze behavioral task to probe the learning, spatial memory and executive function in 9-, 12- and 15-month-old TgF344-AD rats, compared to age-matched NTg littermates to assess the potential cognition sequelae of GABAergic remodeling and neurodegeneration in the presence of ongoing Aβ and tau pathology (Fig. 7). TgF344-AD and NTg rats were assessed for the latency to escape and the complexity of search strategies in the Barnes maze task: a measure of cognitive resources utilized during the task with strategy refinement indicating stronger spatial learning and memory in memory probe tasks, and greater cognitive flexibility and executive function in the reversal trials [24, 29]. Rats learned the Barnes maze task regardless of age or genotype (Additional file 1: Fig. S6). At 9 months, there were no significant differences in the latency to escape or search strategy complexity in the spatial memory probe [*T* = 0.99(28), *P* = 0.33 and Mann–Whitney *U* = 97, *P* = 0.57, respectively; Fig. 7a, b]. However, there was a significant effect of genotype on executive function at 9 months (latency to escape, *F*(1,28) = 10.62, *P* = 0.003) and on cognitive flexibility (search strategy complexity, *F*(1,28) = 14.84, *P* = 0.0006) (Fig. 7c, d). These data indicate that the executive function and cognitive flexibility, but not spatial memory, were impaired in 9-month-old TgF344-AD rats. At 12 months of age, the spatial memory was impaired, with no differences in the latency to escape [*T* = 1.41(24), *P* = 0.17] but significant deficits in search strategy complexity (Mann–Whitney *U* = 42.5, *P* = 0.03; Fig. 7e, f). Surprisingly, at 12 months of age, the TgF344-AD rats exhibited no significant difference in reversal trial performance compared to NTg rats (latency to escape *F*(1,24) = 1.52, *P* = 0.23; search strategy complexity *F*(1,24) = 0.78, *P* = 0.39; Fig. 7g, h). These results were confirmed in a second cohort of 12-month-old rats (reversal trial latency to escape *F(*1,14) = 0.33, *P* = 0.57; and search complexity F(1,14) = 0.66, *P* = 0.43; Additional file 1: Fig. S7).

With increased age, the 15-month-old TgF344-AD rats exhibited significant impairments in the latency to escape, with no significant deficits in search strategy complexity for spatial memory [*T* = 2.41(29), *P* = 0.02 and Mann–Whitney *U* = 75, *P* = 0.08, respectively; Fig. 7i, j]. They also exhibited robust deficits in executive function and cognitive flexibility [latency: *F*(1,29) = 10.08, *P* = 0.004; search strategies: *F*(1,29) = 8.02, *P* = 0.008; Fig. 7k, l]. The spatial memory performance of 15-month-old rats significantly correlated with hippocampal GABAergic neurons (*R*^*2*^ = 0.28, *P* = 0.03), and executive function significantly correlated with PVB (*R*^*2*^ = 0.63, *P* = 0.02), but not SST (*R*^*2*^ = 0.004, *P* = 0.88), dendritic complexity (Additional file 1: Fig. S8).

**Fig. 8:**
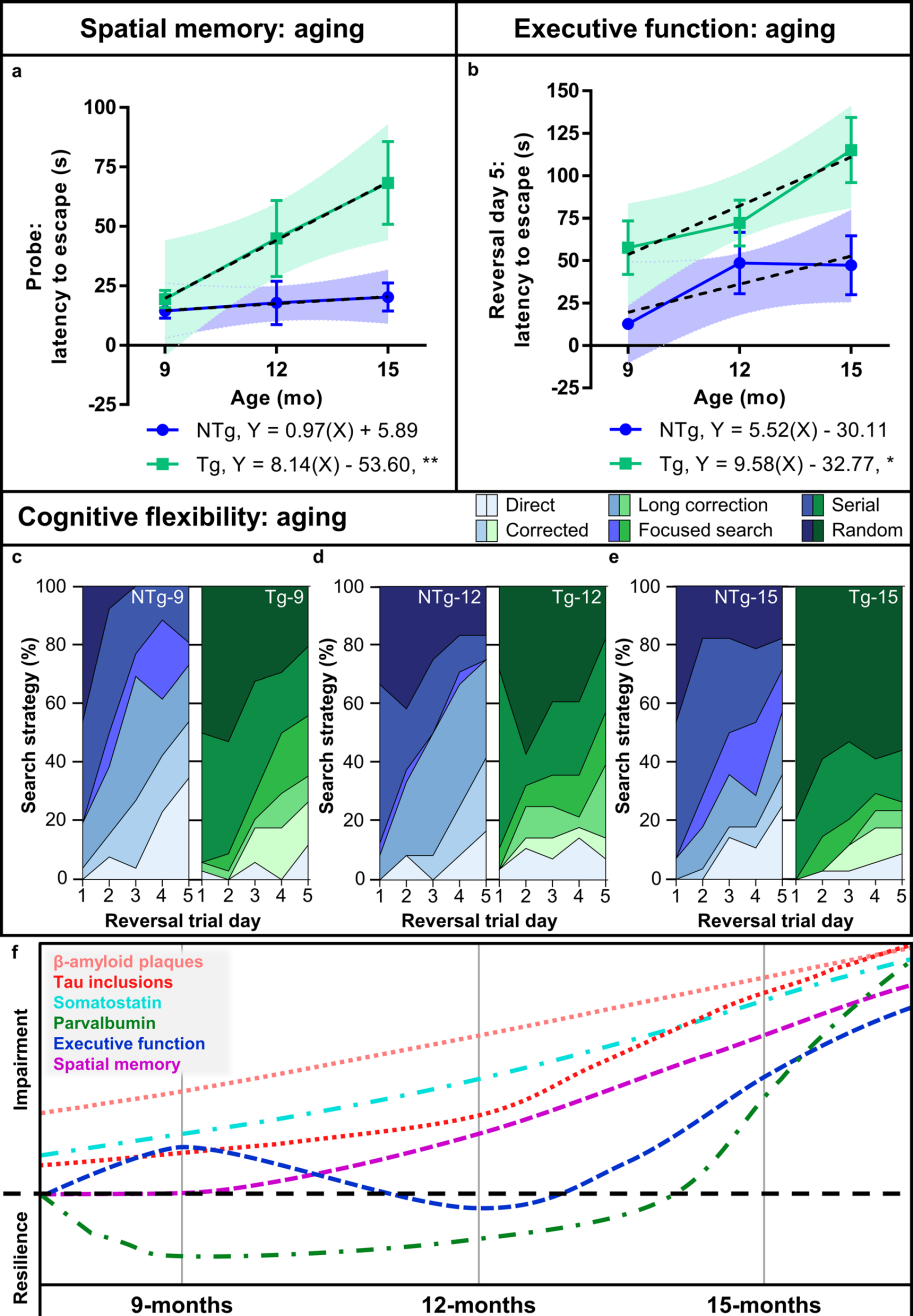
Age affects spatial memory, executive function and cognitive flexibility in TgF344-AD and NTg rats. Barnes maze was conducted in NTg and TgF344-AD rats at 9 (*n* = 13 and 17), 12 (*n* = 12 and 14) and 15 (*n* = 14 and 17) months of age (see Fig. 7). **a**, **b** The latencies to escape in the probe trial and in the final reversal trial day were assessed to determine the effect of aging on spatial memory and executive function. **a** NTg rats did not show significant impairment in spatial memory with age; however, spatial memory dysfunction increased linearly with age in Tg rats. **b** NTg rats exhibited no significant age effect on executive function, despite slight increases in escape latency between 9 and 12 months of age. The Tg rats exhibited significantly increased executive function deficits with age, most prominently between 12 and 15 months. **c–e** Distribution of search strategies utilized in the reversal days demonstrates genotype and age effects on cognitive flexibility. **c** The 9-month-old NTg rats refined their search strategies to a greater degree than Tg rats. **d** At 12 months of age, NTg and Tg rats utilized a comparable distribution of search strategies, with an aging effect in the 9- *vs* 12-month comparison in NTg rats. **e** The 15-month-old NTg rats performed similarly to 12-month-old NTg rats, whereas the 15-month-old Tg rats had robust impairments and were cognitively inflexible, utilizing a majority of random search strategies in reversal trials. **f** A summary schematic demonstrating progressive Aβ and tau accumulation, somatostatin dysfunction and spatial memory deficits, as well as maintenance of executive function associated with a resilient hippocampal parvalbumin network, before robust loss at the endpoint. Data represent mean ± SEM (**a** and **b**; blue and green error bars) and ± 95% confidence intervals (**a** and **b**; blue and green shaded area) of trendline (**a** and **b**; black dashed); linear regression. *P < 0.05, **P < 0.01 for slopes significantly non-zero

We next assessed the effect of age on spatial memory, executive function and cognitive flexibility in TgF344-AD and NTg rats (Fig. 8). The latency to escape in the spatial memory probe trial significantly increased across age in TgF344-AD rats [Y = 8.14 X – 53.60; *F*(1,46) = 7.04, *P* = 0.01], but not in NTg littermates [Y = 0.97 X + 5.89; *F*(1,35) = 0.45, *P* = 0.51; Fig. 8a]. Similarly, the latency to escape in the final reversal trial day significantly increased across age in TgF344-AD rats [Y = 9.58 X − 32.77; *F*(1,46) = 6.37, *P* = 0.02], but not in NTg littermates [Y = 5.52 X − 30.11; *F*(1,35) = 2.33, *P* = 0.14; Fig. 8b]. The spatial memory deficits progressed linearly with age in TgF344-AD rats, while the executive function did not. Interestingly, the executive function remained relatively constant between 9- and 12-months in TgF344-AD rats. To further probe the age effects, we assessed search strategies in the reversal trial as a measure of cognitive flexibility. Search strategies demonstrated a refinement towards use of more complex strategies in 9-month NTg, and to a lesser extent in TgF344-AD rats (Fig. 8c). At 12 months of age, NTg and TgF344-AD rats used comparable search strategies across reversal trials (Fig. 8d). Finally, 15-month-old TgF344-AD rats relied mostly on random search strategies, demonstrating a loss of cognitive flexibility and robust executive function impairment (Fig. 8e). Overall, the TgF344-AD rats exhibited behavioral resilience between 9- and 12-months of age, prior to robust impairment at 15 months of age (Fig. 8c–e).

## Discussion

Cognitive dysfunction in AD occurs as a result of long-term accumulation of Aβ, tau and neurodegeneration in defined regional staging patterns across the brain [1, 25, 43]. However, this linear disease model does not incorporate cognitive reserve, including patient-to-patient variability in cognitive impairment with comparable pathology, presence of Aβ and tau post-mortem in non-demented individuals, and the impact of neuronal compensatory mechanisms [16–19]. Here, we presented a novel discovery of endogenous cognitive resilience in a preclinical AD model. We described a defined stage of resilience of executive function and cognitive flexibility that is reinforced by neuroplastic hippocampal inhibitory remodeling of PVB neurons, and occurs in the presence of spatial memory deficits and continuous Aβ and tau accumulation in the hippocampus and entorhinal cortex. We described remodeling in relation to cognition across disease progression, and proposed that Aβ, tau and neuronal injury impair cognition in 9-month-old TgF344-AD rats, in addition to initiating a compensatory cascade of PVB circuit reorganization. Across disease progression, this reorganization protected against cognitive decline through facilitating recovery of functional GABAergic neuronal marker expression and dendritic sprouting, which were observed in 12-month TgF344-AD rats. Finally, after extensive Aβ and tau accumulation, compensation and resilience are replaced by neurodegeneration and cognitive decline at 15 months of age.

We have previously described disruption of the entorhinal-hippocampal connectivity and accumulation of entorhinal cortical tau, driving spatial memory dysfunction in TgF344-AD rats [24], which is consistent with data from AD patients and other models [2, 44–46]. However, impairment of executive function and cognitive flexibility arises from multiple pathologies [4, 24, 47, 48] and the processes driving cognitive resilience have not been fully elucidated.

Adaptive compensatory changes in hippocampal GABAergic neuronal networks protect against age-related cognitive decline [7, 8], and inhibitory hippocampal remodeling has been demonstrated in AD, including increased GABAergic differentiation in AD patient-derived neuronal cultures [49] and GABAergic sprouting in Aβ-driven mouse models [35, 50]. Hilar GABAergic interneurons drive cognitive impairment in Apolipoprotein E4 (ApoE4) mice via tau-mediated degeneration, in which tau knockout or therapeutic enhancement of GABA signalling rescues the cognitive deficits [51, 52]. The GABAergic changes in ApoE4 versus E3 mice are sex-dependent, with hilar SST loss over age in female, but not male ApoE4 mice, and no ApoE4-related changes in PVB cells in females or males [52]. Although in this study sex differences were not detected in TgF344-AD rats, our results highlight the importance of GABAergic neurons for cognition in AD, and the vulnerability of SST neurons to AD pathology.

Environmental enrichment and experience can modulate GABAergic neuroplasticity and augment excitatory activity to regulate cognitive processes in health and AD [39–41, 48, 53]; however, endogenous inhibitory remodeling in response to AD pathology has been proposed to result in cognitive decline [39, 50]. Our work presents a more dynamic model in which cognitive resilience and maintenance of executive function in AD are reinforced by PVB neuroplasticity, before robust PVB atrophy and cognitive decline.

We postulate that PVB remodeling occurs as a compensatory response to excitatory and inhibitory neuronal damage and loss. Entorhinal cortical afferent connections to the hippocampus are critical in maintaining the excitatory-inhibitory balance via direct innervation of CA1 PVB neurons [54]. Specifically, post-lesion entorhinal cortical neuronal loss can induce compensatory PVB synaptogenesis in the DG, including increased dendritic density [37]; here, we observed a TgF344-AD injury in hippocampus-projecting excitatory neurons in the entorhinal cortex, coinciding with the maximal PVB remodeling in the hippocampus. Furthermore, GABAergic loss and degeneration of SST interneurons in 9-month TgF344-AD rats can cause hyperactivity [11, 55], and decrease the SST-mediated inhibitory gating and integration of excitatory signals at apical dendrites [6]. Our results suggest that endogenous compensatory mechanisms exist between hippocampal GABAergic interneurons, in which the broader, perisomatic-targeted PVB inhibition [6] supplements SST loss. Although we did not directly investigate PVB or SST neuronal function herein, we have previously demonstrated no hippocampal hyperactivity in 9-month TgF344-AD rats with GABAergic cell loss [22], supporting our hypothesis of PVB compensation.

Our results demonstrate an earlier vulnerability of hippocampal SST neurons to tau compared to PVB neurons, and more SST neurons co-localized with tau at the advanced disease state. Our data are consistent with the pathological observation that SST neurons in AD patients were decreased compared to healthy controls, and the majority co-localized with Aβ and tau; whereas PVB neurons were increased and resistant to pathology [56]. Similarly, Blazquez-Llorca and colleagues demonstrated that the hippocampal PVB neurons rarely exhibit intraneuronal paired helical filament tau in AD patients [57]. Finally, loss of SST neurons in AD correlates tightly with Aβ and tau pathology [38, 58], whereas PVB neurons are more resilient [59–61] (reviewed in [12]). Importantly, we demonstrated that the hippocampal PVB neurons in TgF344-AD rats were resistant to tau accumulation, facilitating their compensation for early GABAergic cell loss and SST impairments, and their benefit in cognitive reserve of executive function.

Notably, executive function and cognitive flexibility are correlated with the cognitive reserve in humans [17, 62–64]. Recent evidence suggests that executive function is the prominent cognitive domain represented in cognitive reserve. Computational modeling has demonstrated that variation within cognitive reserve depends on the executive function more so than on memory [62]. Furthermore, executive function measured in the Montreal Cognitive Assessment correlates with and better represents indices of cognitive reserve, including education, occupation and leisure cognitive activity, than the Mini-Mental State Examination which does not test for executive function [63, 64]. This supports our finding of resilient executive function between 9 and 12 months, when there is active decline in spatial memory performance.

We are the first to investigate cognitive reserve in TgF344-AD rats; however, recent evidence supports our study [26, 65]. Voorhees and colleagues [26] demonstrated that the TgF344-AD rats exposed to organophosphate pesticides, an occupational lifestyle risk factor for AD, exhibited cognitive impairments in executive function at 6, 15 and 24 months of age, but not at 9 and 12 months of age. Furthermore, TgF344-AD rats present with a higher magnetic resonance signal in the fiber density-weighted connectome in mid-age, indicative of compensatory hyperconnectivity [65], which is consistent with data of cognitive reserve in mild cognitive impairment patients [66].

## Conclusions

Overall, the GABAergic neuroplasticity in TgF344-AD rats is beneficial in the short-term in which it supports cognitive resilience; however, it is insufficient in the longer-term under extensive accumulation of Aβ and tau pathology, and robust loss of hippocampal excitatory and inhibitory neurons, ultimately leading to significant impairment in spatial memory, executive function and cognitive flexibility. Therefore, we have presented a mechanism of neuronal compensation and cognitive resilience in an AD model, in which PVB neuroplasticity sustains executive function and cognitive flexibility.

## Supplementary Information

**Additional file 1**. **Fig. S1**: GABAergic interneurons in CA1 and CA3 pyramidal layer subregions. **Fig. S2**: Hippocampal and entorhinal cortical area selection for 6F3D and PHF1 analyses. **Fig. S3**: PHF1 co-localization assessments. **Fig. S4**: Parvalbumin neuronal traces in 12- and 15-month-old NTg and TgF344-AD rats. **Fig. S5**: No genotype differences in parvalbumin nor somatostatin dendritic length and complexity in 4-month-old NTg and TgF344-AD rats. **Fig. S6**: NTg and TgF344-AD rats spatial learning in the Barnes maze task. **Fig. S7**: Confirmation of cognitive resilience in executive function in a separate cohort of 12-month-old TgF344-AD rats. **Fig. S8**: GABAergic interneurons and parvalbumin dendritic complexity correlate with spatial memory and executive function, respectively, in 15-month-old rats. **Table S1**: Antibodies and detection kits utilized in this study.

## Data Availability

All data generated or analysed during this study are included in this published article and its supplementary information files. Raw data are available upon reasonable request.

## Abbreviations

Aβ: β-Amyloid
ApoE4: Apolipoprotein E4
DG: Dentate gyrus
GAD67: Glutamate decarboxylase 67
GLS: Glutaminase
NeuN: Neuronal nuclei
NTg: Non-transgenic
PVB: Parvalbumin
SST: Somatostatin
