# Supplementary material for: Parvalbumin neuroplasticity compensates for somatostatin impairment, maintaining cognitive function in Alzheimer’s disease
Source: Transl Neurodegener. 2022 May 3;11:26. doi: 10.1186/s40035-022-00300-6 (PMC9063209; doi:10.1186/s40035-022-00300-6)
Supplement: Supplementary file 1 — Additional file 1. Fig. S1: GABAergic interneurons in CA1 and CA3 pyramidal layer subregions. Fig. S2: Hippocampal and entorhinal cortical area selection for 6F3D and PHF1 analyses. Fig. S3: PHF1 co-localization assessments. Fig. S4: Parvalbumin neuronal traces in 12- and 15-month-old NTg and TgF344-AD rats. Fig. S5: No genotype differences in parvalbumin nor somatostatin dendritic length and complexity in 4-month-old NTg and TgF344-AD rats. Fig. S6: NTg and TgF344-AD rats spatial learning in the Barnes maze task. Fig. S7: Confirmation of cognitive resilience in executive function in a separate cohort of 12-month-old TgF344-AD rats. Fig. S8: GABAergic interneurons and parvalbumin dendritic complexity correlate with spatial memory and executive function, respectively, in 15-month-old rats. Table S1: Antibodies and detection kits utilized in this study. [file 40035_2022_300_MOESM1_ESM.pdf]

Parvalbumin neuroplasticity compensates for somatostatin impairment, maintaining cognitive function in Alzheimer's disease

Supplementary information

Index

Figure S1 GABAergic interneurons in CA1 and CA3 pyramidal layer subregions. (page 2)

Figure S2 Hippocampal and entorhinal cortical area selection for 6F3D and PHF1 analyses (page 3)

Figure S3 PHF1 co-localization assessments. (page 4)

Figure S4 Parvalbumin neuronal traces in 12- and 15-month-old NTg and TgF344-AD rats. (page 5)

Figure S5 No genotype differences in parvalbumin nor somatostatin dendritic length and complexity in 4-month-old NTg and TgF344-AD rats. (page 6)

Figure S6 NTg and TgF344-AD rats spatial learning in the Barnes maze task. (page 7)

Figure S7 Confirmation of cognitive resilience in executive function in a separate cohort of 12-month-old TgF344-AD rats. (page 8)

Figure S8 GABAergic interneurons and parvalbumin dendritic complexity correlate with spatial memory and executive function, respectively, in 15-month-old rats. (page 9)

Table S1 Antibodies and detection kits utilized in this study. (page 10)

Supplementary figures

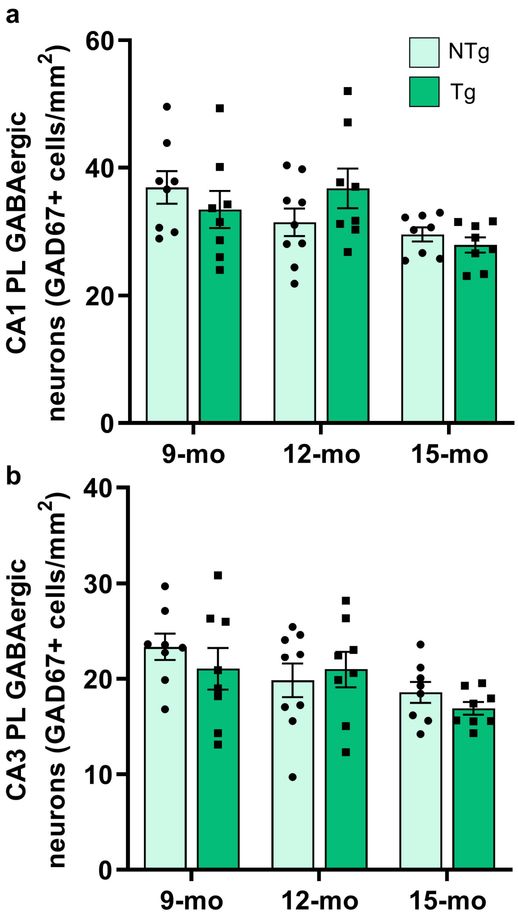

Figure S1: GABAergic interneurons in CA1 and CA3 pyramidal layer subregions. In 9-, 12- and 15-month-old NTg and TgF344-AD rats (n = 8-9), GABAergic interneurons were assessed by GAD67 staining and quantified within the hippocampus (see **Fig. 2**). (**a**) In the CA1 pyramidal layers (PL), there was an overall significant effect of age [$F(2,43) = 4.42$, $P = 0.02$], yet no difference by genotype [$F(1,43) = 0.001$, $P = 0.97$] or age X genotype interaction [$F(2,43) = 2.05$, $P = 0.14$]. (**b**) Similarly, in the CA3 PL there was an overall significant effect of age [$F(2,43) = 3.90$, $P = 0.03$], with no significant effect of genotype [$F(1,43) = 0.51$, $P = 0.48$] or age X genotype interaction [$F(2,43) = 0.67$, $P = 0.52$]. Data are mean ± SEM; two-way ANOVA.

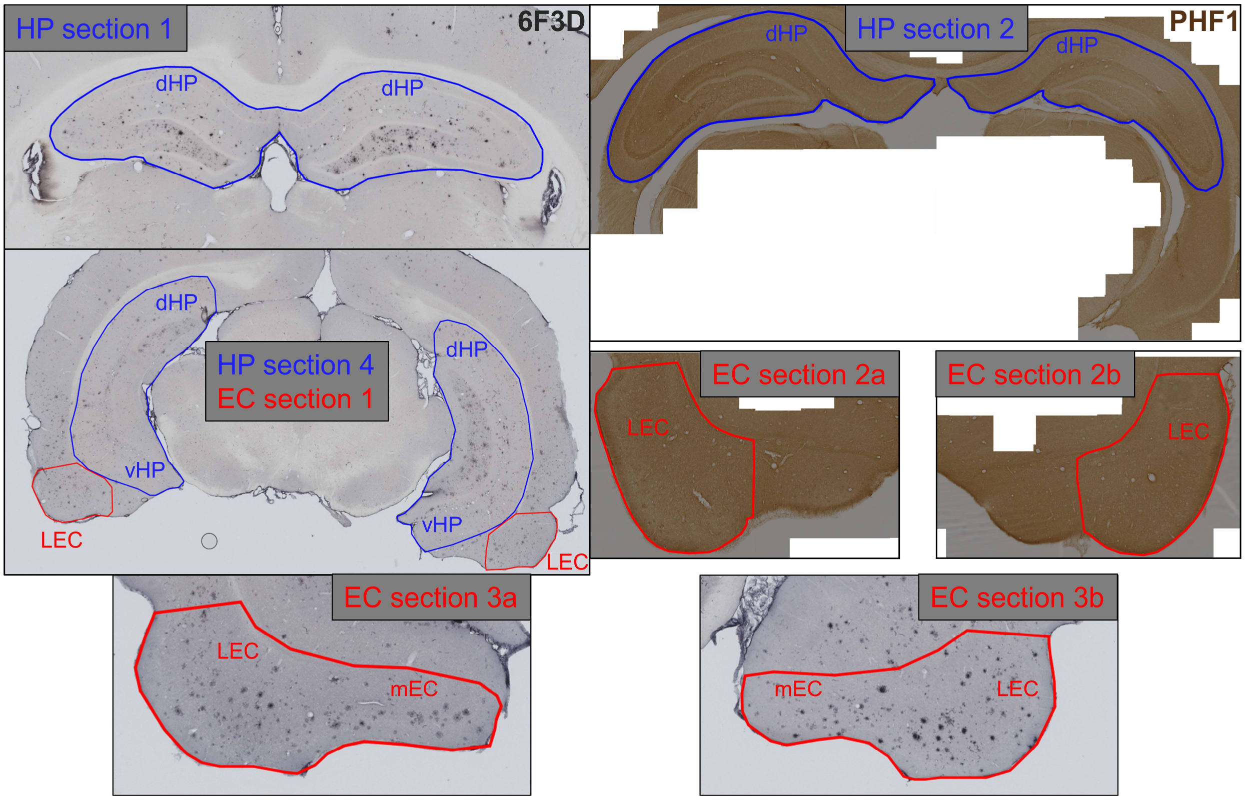

Figure S2: Hippocampal and entorhinal cortical area selection for 6F3D and PHF1 analyses. Immunohistochemistry with 6F3D and PHF1 was conducted to assess TgF344-AD Aβ and tau pathology, respectively, in the hippocampus (HP) and entorhinal cortex (EC; see **Fig. 3**). Four HP sections and three EC sections were quantified for 6F3D analysis, and three hippocampal sections and three entorhinal cortical sections for PHF1 analysis. Representative areas included in these analyses are depicted; blue selection for HP. red selection for EC. Area of analysis included dorsal and ventral HP (dHP/vHP), and lateral and medial EC (LEC/mEC). Images were captured at 10x magnification. Images are not to scale.

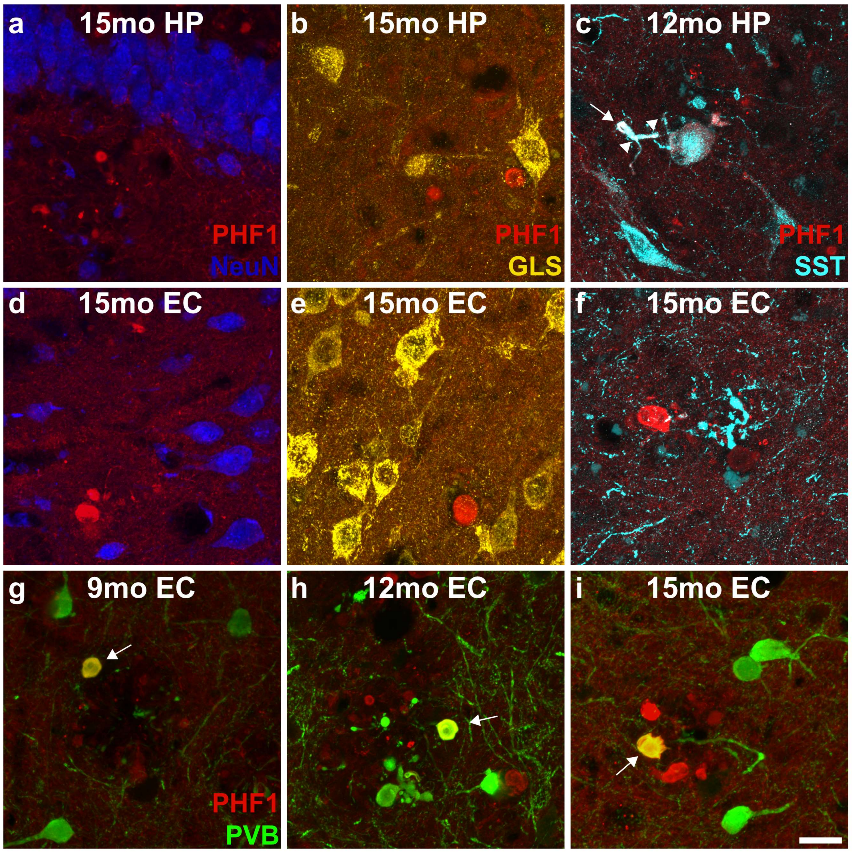

Figure S3: PHF1 co-localization assessments. Continuation of data from **Fig. 4**: to determine the effect of tau pathology on excitatory and inhibitory neurons in the hippocampus (HP) and entorhinal cortex (EC) of TgF344-AD rats. PHF1 co-localization with four neuronal markers was assessed via immunofluorescent staining (representative images from *n* of 6-12 sections/group). (**a and b**) In the HP, neither NeuN or glutaminase (GLS) show tau deposits at 9-, 12- and 15-months of age (representative 15-month images are shown). NeuN is a pan-neuronal marker, but lack of obvious co-localization in cell layers (dense with excitatory neurons) indicates lack of tau inclusions in excitatory neurons of TgF344-AD rats. GLS is an excitatory-specific neuronal marker. HP SST interneurons display cytoplasmic tau inclusions as early as 9-months (see **Fig. 4**), and demonstrated here (**c**) with tau inclusions in the cytoplasm (white arrow) and processes (white arrowheads) as early as 12-months. (**d and e**) In the EC, neither NeuN or GLS show tau deposits at 9-, 12- and 15-months of age (representative 15-month images are shown). (**f**) EC SST interneurons also do not co-localize with PHF1 at 9-, 12- or 15-months of age (representative 15-month image is shown), but appear to surround plaques and demonstrate neurodegenerative morphology as in the HP (see **Fig. 4c**). (**g-i**) EC PVB interneurons co-localize with PHF1 (cytoplasmic inclusions; white arrows) at 9-, 12- and 15-months of age. Scale bar represents 20 μm.

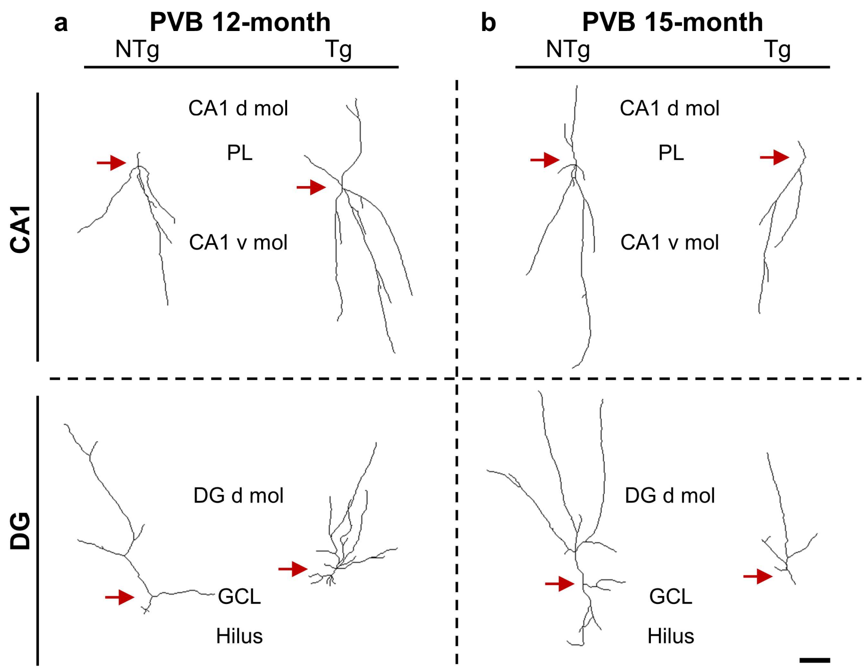

Figure S4: Parvalbumin neuronal traces in 12- and 15-month-old NTg and TgF344-AD rats. PVB neuronal tracing and Sholl analysis was conducted in NTg and TgF344-AD rats at 9-, 12- and 15-months of age (see **Fig. 5** and **Fig. 6i-p**). Representative PVB neuronal traces demonstrate increased dendritic length and complexity in the Tg CA1 and DG at 9- (see **Fig. 6i and m**) and 12-months of age (**a**), and decreased dendritic length and complexity at 15-months (**b**), compared to NTgs. Scale bar represents 50 µm. Abbreviations: dorsal/ventral molecular layer (d/v mol); granular cell layer (GCL); pyramidal layer (PL).

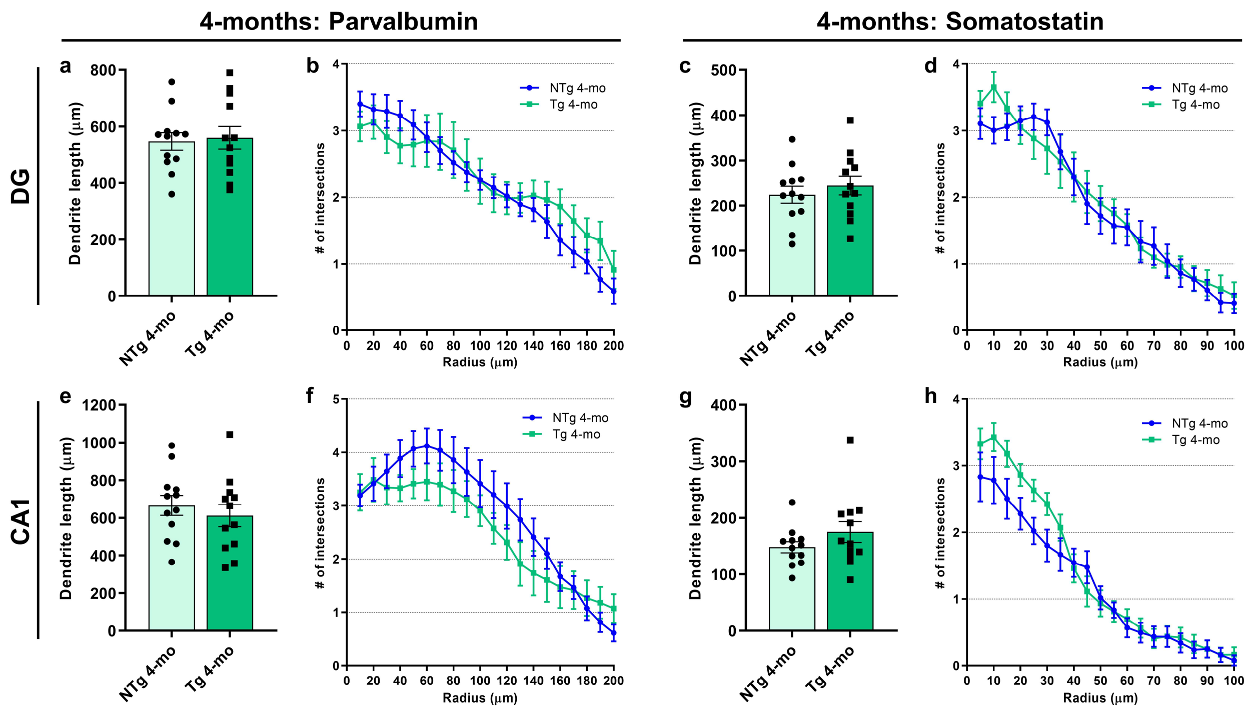

Figure S5: No genotype differences in parvalbumin nor somatostatin dendritic length and complexity in 4-month-old NTg and TgF344-AD rats. We probed the TgF344-AD hippocampal GABAergic network via neuronal tracing of parvalbumin (PVB) and somatostatin (SST) neurons, to assess total dendritic length and complexity of intersections with Sholl analysis (see **Figs. 4-6**). PVB and SST neurons were assessed in 4-month-old NTg and TgF344-AD rats to determine if 9-month Tg alterations are present at this pre-pathology stage. (**a and b**) In the dentate gyrus (DG), no significant differences were detected in PVB dendritic length ($T = 0.26(22)$, $P = 0.80$) or number of intersections [genotype: $F(1,22) = 0.12$, $P = 0.74$; genotype X distance interaction: $F(19,418) = 1.09$; $P = 0.36$]. (**c and d**) Similarly, DG SST neurons in NTg and Tg rats did not differ in length ($T = 0.73(22)$, $P = 0.47$) or complexity [genotype: $F(1,22) = 0.07$, $P = 0.80$; genotype X distance interaction: $F(19,418) = 0.67$, $P = 0.85$]. No NTg-Tg differences were detected in CA1 (**e and f**) PVB [length, ($T = 0.69(22)$, $P = 0.50$); complexity, genotype: $F(1,22) = 1.26$, $P = 0.27$; genotype X distance interaction: $F(19,418) = 1.02$, $P = 0.44$] and (**g**) SST neuronal length ($T = 1.29(22)$, $P = 0.21$). (**h**) There were no significant effects of genotype [$F(1,22) = 2.40$, $P = 0.14$] or genotype X distance interaction [$F(19,418) = 1.57$, $P = 0.06$] in CA1 SST complexity. Data are mean ± SEM (from n of 12 cells/cohort, sampled across 3 rats/genotype); unpaired t-test (**a,c,e and g**) or repeated measures ANOVA (**b,d,f and h**).

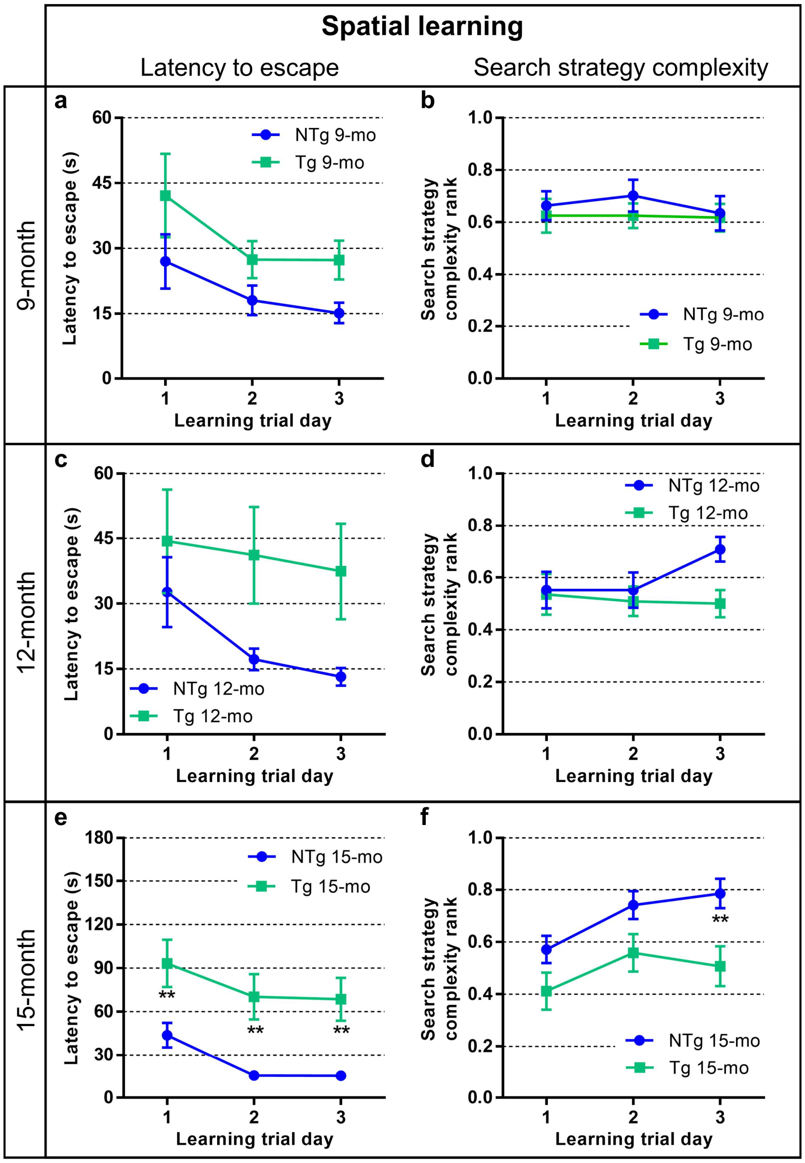

Figure S6: NTg and TgF344-AD rats spatial learning in the Barnes maze task. Before the spatial memory probe and reversal learning (see **Fig. 7**) in the Barnes maze, NTg and TgF344-AD rats were tested in 3 trial days to assess learning of the escape hole location. (**a-d**) At 9- and 12-months, Tg rats display significant changes and no differences, respectively, in overall performance in the latency to escape [(**a**) $F(1,28) = 5.01$, $P = 0.03$ and (**c**) $F(1,24) = 3.77$, $P = 0.06$, respectively], but had no significant differences on specific trial days or in search strategy complexity [(**b**) $F(1,28) = 0.61$, $P = 0.44$ and (**d**) $F(1,24) = 1.64$, $P = 0.21$, respectively]. At 15-months, Tg rats are significantly impaired in spatial learning latency to escape [(**e**) $F(1,29) = 10.45$, $P = 0.003$] and search strategy complexity [(**f**) $F(1,29) = 6.80$, $P = 0.01$], with significant differences across all 3 trial days. Overall, these data demonstrate that Tg rats exhibit subtle deficits in spatial learning across disease progression. 9-months ($n = 13$ and 17); 12-months ($n = 12$ and 14); 15-months ($n = 14$ and 17). Data are mean ± SEM; repeated measures ANOVA with Holm-Sidak *post hoc* test; $^{**}P < 0.01$.

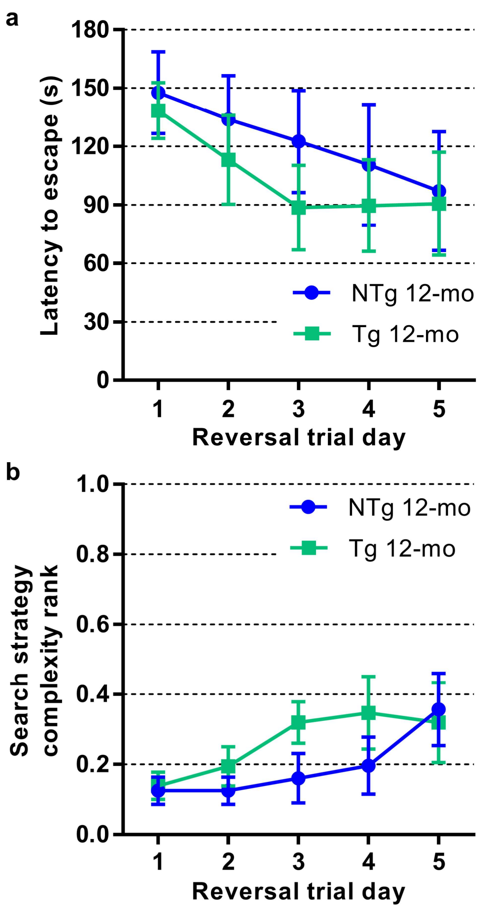

Figure S7: Confirmation of cognitive resilience in executive function in a separate cohort of 12-month-old TgF344-AD rats. A second cohort of 12-month NTg (n = 7) and TgF344-AD (n = 9) rats were tested in the Barnes maze to confirm cognitive resilience in executive function and cognitive flexibility. Similar to the results in **Fig. 7**, no genotype differences were detected at 12-months in the latency to escape [(**a**) $F(1,14) = 0.33$, $P = 0.57$] or search strategy complexity [(**b**) $F(1,14) = 0.66$, $P = 0.43$] in the reversal trials. Data are mean ± SEM; repeated measures ANOVA with Holm-Sidak *post hoc* test.

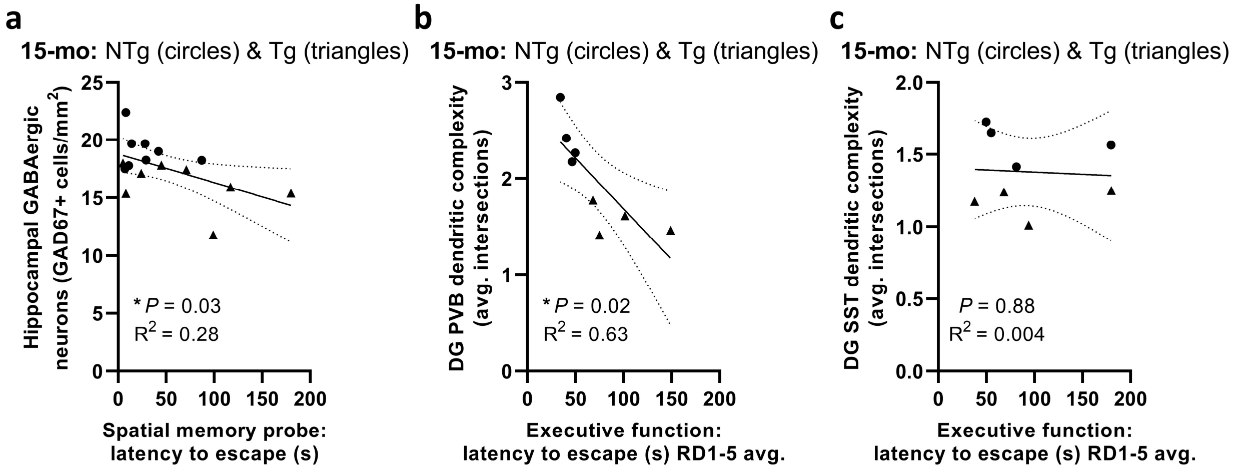

Figure S8: GABAergic interneurons and parvalbumin dendritic complexity correlate with spatial memory and executive function, respectively, in 15-month-old rats. Cognitive performance in the Barnes maze (see **Fig. 7**), was assessed for correlation with hippocampal GABAergic neurons (see **Fig. 2**), and parvalbumin (PVB) and somatostatin (SST) dendritic complexity (see **Fig. 6**). (**a**) In 15-month-old NTg (circles) and TgF344-AD (triangles) rats, a significant negative relationship was detected between spatial memory latency to escape, and the number of hippocampal GABAergic neurons (*n* = 16 rats, Y = -0.03*X + 18.77, $R^2 = 0.28$, $F(1,14) = 5.51$, $P = 0.03$). (**b and c**) In 15-month old rats, executive function performance (average latency to escape over 5 days) significantly correlated with PVB (*n* = 8 rats, Y = -0.01*X + 2.74, $R^2 = 0.63$, $F(1,6) = 10.21$, $P = 0.02$), but not SST (*n* = 8 rats, Y = -0.0003*X + 1.41, $R^2 = 0.005$, $F(1,6) = 0.03$, $P = 0.88$), dendritic complexity [average intersections < 200 μm (PVB) or < 100 μm (SST) from cell soma]. Quicker completion of the spatial memory task associated with a greater number of GABAergic neurons, and of the executive function trials with greater PVB dendritic complexity, in 15-month-old rats. No correlations were detected at other ages. Data are mean ± 95% confidence intervals (dotted lines); linear regression; *$P < 0.05$.

Supplementary tables

Table S1: Antibodies and detection kits utilized in this study.

Antibodies and Detection Kits	Source	Identifier
Mouse monoclonal anti-human amyloid-beta peptide (6F3D)	Dako, Agilent	Cat#M0872, RRID:AB_2056966
Mouse monoclonal IgG1 anti-PHF1	Courtesy of Peter Davies	N/A
Mouse monoclonal anti-GAD67, clone 1G10.2	Millipore Sigma	Cat#MAB5406, RRID:AB_2278725
Guinea pig polyclonal anti-NeuN	Millipore Sigma	Cat#ABN90, RRID:AB_11205592
Rabbit polyclonal anti-SST	Invitrogen, Thermo Fisher Scientific	Cat#PA5-85759, RRID:AB_2792896
Mouse monoclonal anti-PVB (for neuronal tracing and sholl analysis)	Swant	Cat#PV 235
Rabbit polyclonal anti-PVB (for PHF1 co-localization assessment)	Abcam	Cat#ab11427, RRID:AB_298032
Rabbit polyclonal anti-KGA/GAC (GLS)	Proteintech	Cat#12855-1-AP, RRID:AB_2110381
Goat polyclonal anti-mouse IgG1 biotin-XX	Invitrogen, Thermo Fisher Scientific	Cat#A10519, RRID:AB_2534028
Donkey polyclonal anti-guinea pig Alexafluor594	Jackson ImmunoResearch Laboratories	Cat#706-585-148, RRID:AB_2340474
Donkey polyclonal anti-mouse Alexafluor488	Invitrogen, Thermo Fisher Scientific	Cat#A-21202, RRID:AB_141607
Donkey polyclonal anti-rabbit Alexafluor488	Invitrogen, Thermo Fisher Scientific	Cat#A-21206, RRID:AB_2535792
Streptavidin Alexafluor647	Invitrogen, Thermo Fisher Scientific	Cat#S21374
Vectastain ABC Kit – polyclonal horse anti-mouse IgG biotin	Vector Laboratories	Cat#PK-4002, RRID:AB_2336811
3,3'-diaminobenzidine peroxidase substrate kit	Vector Laboratories	SK-4100, RRID:AB_2336382

Abbreviations: glutamate decarboxylase 67 (GAD67); glutaminase (GLS); neuronal nuclei (NeuN); parvalbumin (PVB); research resource identifier (RRID); somatostatin (SST).
